# Development of a predictive model for meglumine antimoniate treatment failure in patients with cutaneous leishmaniasis: Aretrospective cohort study

**DOI:** 10.1371/journal.pntd.0014606

**Published:** 2026-08-04

**Authors:** Susana Ríos-Echavarría, Gilma Hernández Herrera, Hector Ivan García García, Liliana López-Carvajal, Lina Maria Serna-Higuita

**Affiliations:** 1 Group of Clinical Trials PECET, University of Antioquia, Medellín, Colombia; 2 Academic Group of Clinical Epidemiology (GRAEPIC), University of Antioquia, Medellín, Colombia; 3 Department of Clinical Epidemiology and Applied Biostatistics, University Hospital Tübingen, Tübingen, Germany; Universidade do Estado do Rio de Janeiro, BRAZIL

## Abstract

**Background:**

Although alternative therapies for cutaneous leishmaniasis (CL) are available, systemic meglumine antimoniate (MA) remains the first-line treatment in many endemic regions. Its use is nevertheless associated with serious adverse effects and a high risk of treatment failure (TF). This study aimed to identify clinical and sociodemographic risk factors for TF following systemic MA therapy, which may enhance therapeutic decision-making and improve clinical outcomes.

**Methodology:**

We evaluated a retrospective cohort of 296 patients with CL treated with MA between 2007 and 2024. A multivariable logistic regression model was performed using candidate variables selected via clinical relevance, biological plausibility, stepwise selection and least absolute shrinkage and selection operator (Lasso) regression. Model performance was assessed through discrimination, calibration, and internal validation. Results were reported as odds ratios, 95% confidence intervals and p-values.

**Principal Findings:**

All included patients received first line MA therapy and completed at least six months of follow-up. Independent predictors associated with TF were age, occupational activity, size, number and anatomical location of lesions, clinical form, regional lymphadenopathy, and prior history of leishmaniasis. The final model showed moderate overall performance with a Hosmer-Lemeshow p value = 0.667, AUC = 0.691, and a Brier score of 22.8. Internal validation yielded a Harrel C = 0.608.

**Conclusions:**

The present study demonstrates an association between TF and socio-demographic, clinical variables. Identifying these risk factors may support clinical decision-making and contribute to optimizing treatment outcomes.

## Author summary

Cutaneous leishmaniasis is a parasitic disease that causes skin lesions that remains an important health problem in many parts of the world. Although alternatives therapies exist, systemic meglumine antimoniate remains the most used treatment in many countries. However, its clinical utility is frequently limited by severe adverse effects and a high risk of therapeutic failure we evaluated a historical cohort of patients treated with meglumine antimoniate between 2007 and 2024 to identify predictors of treatment failure. Our findings show that treatment was less successful in patients who were younger, living in rural place, prolonged disease duration, with multiple lesions, and prior history of leishmaniasis. Recognizing these factors can guide clinician in selecting optimized treatments and improve care for people with cutaneous leishmaniasis.

## Introduction

Leishmaniasis is a zoonotic disease caused by parasites of the genus *Leishmania*, and transmitted by *Lutzomyia* vector of the subfamily *Phelobominae* [1,2]. In Colombia, around 11 million people are at risk of Leishmaniasis infection, with rural workers with low socioeconomic conditions being the most affect. This population additionally faces health care barriers which limit the access to early diagnosis and treatment availability [3–7]. Among the various clinical manifestations, cutaneous leishmaniasis (CL) is the most prevalent form [8], with an incidence of 18.6 cases per 100,000 inhabitants in 2022 [9].

Systemic meglumine antimoniate (MA) has been used as first-line treatment in patients with CL for over 70 years. However, its use is related with a wide range of side effects [2], including cardiac, renal, hepatic and pancreatic toxicity [10,11]. MA has been also associated with an increasing risk of therapeutic failure (TF), with an estimated prevalence of 23.4% across Latin America [12–14]. Previous studies have identified several factor associated with TF like age, [13,15,16], lesion size number [17] and duration of skin lesion [12], *Leishmania* species [2,13,17–20], geographical location [2], compliance therapy [12], presence of lymphadenopathies [12], weight and previous history of leishmaniasis [20,21].

The Pan American Health organization (PAHO) currently recommends classifying CL treatment as local or systemic based on lesion characteristics and the feasibility of patient follow up [22]. Recent studies has reported adequate cure rates with the use of miltefosine as the first systemic treatment option in patients with CL [23]. However, MA remains widely used in our Colombia due to political and logistical difficulties, as well as the lack of knowledge about the infection among healthcare professionals [24].

Identifying socio-demographic, clinical, and environmental risk factors for TF can improve clinical decision-making and patient outcomes in CL. A predictive model in Peru using polymerase chain reaction (PCR)-based *Leishmania* species identification showed high accuracy, however, that study excluded non-ulcerated or mixed lesions and which often have higher parasite loads and may be linked to TF [18]. Additionally, PCR is not widely accessible in primary healthcare settings, limiting the model’s practical utility [18]. Our study aimed to identify risk factors related to TF to MA, using variables easily collected by healthcare providers. Which enables the development of a predictive risk model to support clinical decision-making and optimize therapy selection for patients diagnosed with CL.

## Materials and methods

### Ethics statements

This study was conducted in accordance with the ethical principles of the Declaration of Helsinki and the International Council for Harmonization Good Clinical Practice Guidelines. The study protocol was reviewed and approved by the Review Board (IRB) approval of the University of Antioquia. As part of their routine medical care, all patients provided written informed consent that included authorization for the secondary use and analysis of their clinical data for research purposes. Any such secondary use, including future related research or pooled analyses, complies with Institutional ethics standards and relevant data protection regulations. To ensure participant confidentiality and privacy, all data were fully anonymized prior to analysis, and no identifiable personal information was accessed or stored.

### Study design and population

This single-center retrospective cohort analysis included patients with diagnosed with CL who were treated at PECET (Programa de Estudio y Control de Enfermedades Tropicales, Antioquia University, Medellín - Colombia) between 2007 and 2024. All patients received MA (20 mg/kg/day) as first-line therapy for up to 20 days. Patients with mucosal or visceral Leishmaniasis, those who did not agree to sign the informed consent and patients with post treatment follow-up less than 3 months were excluded.

### Data collection

Socio-demographic, clinical, and post treatment follow up information was extracted from the electronic health records of PECET. Sociodemographic variables included age, gender, economic activity, place of residence and comorbidities. Clinical variables comprised the site of CL infection, duration of skin lesion in days, number and lesion size (area). For ulcerated lesions, the area was calculated using the rhomboid formula (major diagonal × minor diagonal/ 2), whereas for non-ulcerated lesions, the area was calculated using the rectangle formula (base × height). Additional variables included anatomical location of lesions, presence of regional lymphadenopathy, and history of previous leishmaniasis episodes. Occupational activity was categorized as high-risk occupations—including military personnel and agricultural workers—and low-risk occupations—including individuals with higher education, independent workers such as drivers, miners, self-employed individuals, and construction workers. Adherence to treatment was defined as receiving at least 90% of the prescribed regimen, corresponding to a complete treatment dose of 20 mg/kg/day (maximum 15 ml per day) for 18 days. Adherence was evaluated at the end-of-treatment visit, during which patients returned all medication blisters to their physician for verification.

### Outcomes

The primary outcome of this study was TF to MA, defined as the presence of at least one of the following criteria three months post treatment: 1) a ≥ 50% increase in lesion size, 2) lesion progression after initial regression (including enlargement of a plaque, nodule, or ulcer), or 3) incomplete re-epithelialization and/or persistent inflammatory signs such edema or erythema [9,25].

### Sample size estimation

Following the recommendation proposed by Riley et al., [18,25,26], an assuming an R^2^ Nagelkerke of 0.54 derived from a previous prognostic model (estimated by the Cox-Snell R^2^) [18,25,26], a shrinkage factor of 0.9, a TF prevalence of 23.4% [12–14], and a maximum of 12 candidate predictors. The required sample size was estimated to be 286 participants.

### Statistical analysis

Continuous variables were summarized as mean and standard deviation (SD) or median and interquartile range (IQR) depending on their distribution. The distribution of continuous variables was assessed using kurtosis, skewness, Q-Q plots, and histograms. Categorical variables were reported as absolute and relative frequency.

Variable selection was guided by univariate binary logistic regression model and clinical reasoning [26,27]. Additionally, least absolute shrinkage and selection operator (Lasso) regression model was used to identify relevant predictors by shrinking coefficients toward zero [26,28,29]. Multivariable binary logistic regression models were carried out based on the selected variables. A total of five models were evaluated before selecting the final model. The linearity relationship between continuous predictors with the log-odds of the outcome was evaluated using spline regression models [30] (S1 Fig). Multicollinearity among predictors was assessed using the variance inflation factor (VIF) [31]. Results are reported as crude (univariate regression model) and adjusted (multiple regression model) odds-ratios (OR) with their corresponding 95% confidence intervals (CI). Based on the final model, a risk score was developed. Each variable included in the final model was assigned a weight according to its beta coefficient, which was multiplied by 10 and rounded to the nearest integer to obtain the corresponding point value.

Model fitting was evaluated using the Hosmer-Lemeshow [34,35], R^2^ Nagelkerke, Akaike information criterion (AIC) and the chi-square goodness of fit statistic [32]. Discrimination was evaluated by calculating the receiver operator characteristics (ROC) and the concordance Harrell’s C-index [26,30]. Calibration was evaluated using calibration curves and Brier scores [30]. Internal validation of the final predictive model was conducted via bootstrapping with 1,000 resamples [27].

Missing data were assumed to be at random [27] and multiple chained equation imputation was used to replace lost data with plausible values, based on the observed data. Fifty imputations were implemented and the estimates were pooled according to Rubin’s Rules [33,34]. For all analyses, a 2-tailed *P* value of <0.05 was considered statistically significant. SPSS software (version 28.0; IBM, Chicago, IL) and R (version 4.3) software were used.

## Results

**Fig 1:**
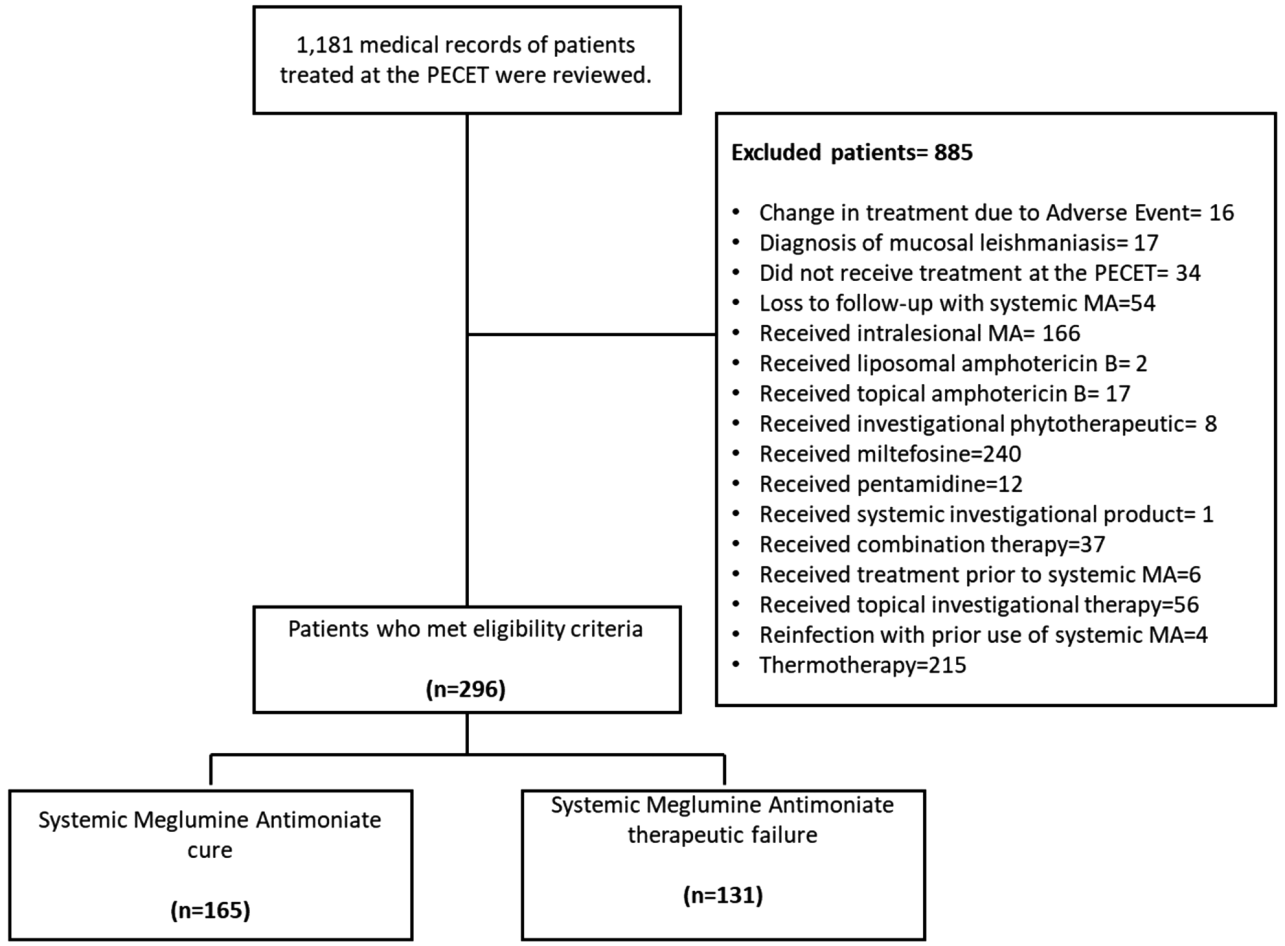
Patient selection flow chart.

A total of 1181 patients were evaluated between 2007 and 2024. Of these, 885 patients were excluded for reasons outlined in Fig 1. A total of 296 patients were included in this study. The baseline characteristics of the cohort are presented in Table 1. Among 296 enrolled patients, 44.2% (n = 131) had TF. The mean age was 26.2 years (±12.2 years, min – max: 0.5 – 66 years), with males predominating at 86.8%. CL was frequently diagnosed via direct parasitological examination (273/296 patients), whereas only 28 patients underwent PCR to identify the infectious species. When comparing patients with and without TF, differences were observed in age (p = 0.012), and duration of skin lesion (p = 0.029). Regarding occupational activity, individual working in professional fields (Administrative, social, human health, and animal health sciences fields) exhibited a trend toward higher cure rates (p = 0.066). No differences were found regarding gender, weight, geographic area of infection, comorbidities, number of lesions, size of the largest lesion, or anatomical location of lesions. The majority of patients resided in rural areas (95.5%), with the Andina, Orinoco, and Amazonia regions accounting for the highest number of patients with systemic MA for CL.

**Table 1:** Baseline characteristics stratified according to the presence of TF to systemic MA.

	Cure (N = 165)	Therapeutic failure (N = 131)	Total (N = 296)	P value
**Age (years)**				
mean (SD)	27.8 (12.3)	24.2 (11.9)	26.2 (12.2)	0.012^TT^
Min – max	3 – 66	0.5 – 64	0.5 - 66	
Age less 18 years	18 (10.9%)	20 (15.3%)	38 (12.8%)	
**Gender**				
Male n (%)	141 (85.5%)	116 (88.5%)	257 (86.8%)	0.542^Chi^
Female n (%)	24 (14.5%)	15 (11.5%)	39 (13.2%)	
**Weight** (Kg)				
(SD)	67.6 (14.4)	65.1 (20.3)	66.6 (17.2)	0.254^TT^
*Missing values*	8 (4.8%)	10 (7.6%)	18 (6.1%)	
**Economic activity** ^*α*^				
military personnel n (%)	76 (46.1%)	71 (54.2%)	147 (49.7%)	0.066^Chi^
Agriculture n (%)	24 (14.5%)	19 (14.5%)	43 (14.5%)	
Independent worker n (%)	13 (7.9%)	9 (6.9%)	22 (7.4%)	
Higher education n (%)	18 (10.9%)	3 (2.3%)	21 (7.1%)	
Others n (%)	34 (20.6%)	29 (22.1%)	63 (21.3%)	
**Place of residence**				
Rural n (%)	156 (94.5%)	123 (96.9%)	279 (95.5%)	0.403^Fi^
Urban n (%)	9 (5.5%)	4 (3.1%)	13 (4.5%)	
**Geographic regions**				
Amazon n (%)	43 (26.2%)	44 (33.8%)	87 (29.6%)	0.430^Fi^
Andean n (%)	66 (40.2%)	51 (39.2%)	117 (39.8%)	
Caribbean n (%)	5 (3.0%)	2 (1.5%)	7 (2.4%)	
Orinoquia n (%)	29 (17.7%)	23 (17.7%)	52 (17.7%)	
Pacific n (%)	21 (12.8%)	10 (7.7%)	31 (10.5%)	
*Missing values*	1 (0.6%)	1 (0.8%)	2 (0.7%)	
**LC diagnosis**				
Biopsy n (%)	10 (6.1%)	1 (0.8%)	11 (3.8%)	0.0443^Fi^
Culture n (%)	1 (0.6%)	2 (1.6%)	3 (1.0%)	
Direct n (%)	152 (92.7%)	121 (96.8%)	273 (94.5%)	
IDR n (%)^b^	1 (0.6%)	1 (0.8%)	2 (0.7%)	
*Missing values*	1 (0.6%)	6 (4.6%)	7 (2.4%)	
***Leishmania* spp.** ^*c*^				
*L.braziliensis n (%)*	0 (0%)	1 (8.3%)	1 (3.6%)	
*L.panamensis n (%)*	16 (100%)	11 (91.7%)	27 (96.4%)	
*Missing values*	149 (90.3%)	119 (90.8%)	268 (90.5%)	
**Comorbidities** ^*d*^				
Yes n (%)	15 (9.1%)	8 (6.1%)	23 (7.8%)	0.3881^Chi^
No n (%)	150 (90.9%)	123 (93.9%)	273 (92.2%)	
**Duration of skin lesion in days**				
Median (RIQ)	60 (42- 91)	56 (30- 84)	56 (31.5- 90)	0.029^MW^
*Missing values*	8 (4.8%)	6 (4.6%)	14 (4.7%)	
**Size of lesion (mm**^**2**^) ^**e**^				
Median (RIQ)	177.9 (47.12 - 588.70)	148.83 (39.25- 462.55)	171.17 (43.79- 500.50)	0.493^MW^
*Missing values*	11 (6.7%)	25 (19.1%)	36 (12.2%)	
**Number of lesions**				
1 n (%)	90 (55.2%)	81 (62.8%)	171 (58.6%)	0.506^Chi^
2 n (%)	26 (16%)	20 (15.5%)	46 (15.8%)	
3 n (%)	15 (9.2%)	11 (8.5%)	26 (8.9%)	
4 n (%)	9 (5.5%)	7 (5.4%)	16 (5.5%)	
5 or more n (%)	23 (14.1%)	10 (7.8%)	33 (11.3%)	
*Missing values*	2 (1.2%)	2 (1.5%)	4 (1.4%)	
**Anatomical location of lesions**				
Head and neck n (%)	24 (14.9%)	25 (19.7%)	49 (17%)	0.428^Chi^
Upper limbs n (%)	62 (38.5%)	43 (33.9%)	105 (36.5%)	
Lower limbs n (%)	22 (13.7%)	25 (19.7%)	47 (16.3%)	
Trunk n (%)	14 (8.7%)	9 (7.1%)	23 (8%)	
More than one lesion n (%)	39 (24.2%)	25 (19.7%)	64 (22.2%)	
*Missing values*	4 (2.4%)	4 (3.1%)	8 (2.7%)	
**Clinical lesion type** ^*f*^				
Ulcerated n (%)	133 (83.1%)	110 (84.6%)	243 (83.8%)	0.716^Chi^
Non-ulcerated n (%)	9 (5.6%)	9 (6.9%)	18 (6.2%)	
Mixed n (%)	18 (11.3%)	11 (8.5%)	29 (10%)	
*Missing values*	5 (3.0%)	1 (0.8%)	6 (2.0%)	
**Presence of local adenopathy**				
Yes n (%)	46 (33.1%)	31 (27.7%)	77 (30.7%)	0.431^Chi^
No n (%)	93 (66.9%)	81 (72.3%)	174 (69.3%)	
*Missing values*	26 (15.8%)	19 (14.5%)	45 (15.2%)	
**Doses of AM** (20 mg/kg/day) ^*g*^				
Insufficient n (%)	2 (1.2%)	5 (3.9%)	7 (2.4%)	0.231^Chi^
Sufficient n (%)	149 (91.4%)	111 (86%)	260 (89%)	
High doses n (%)	12 (7.4%)	13 (10.1%)	25 (8.6%)	
*Missing values*	2 (1.2%)	2 (1.5%)	4 (1.4%)	
**Adherence** ^*h*^				
< 90%	0 (%)	5 (3.8%)	5 (1.7%)	0.016^Fi^
≥ 90%	165 (100%)	126 (96.2%)	291 (98.3%)	
**Prior leishmaniasis infection**				
Yes n (%)	31 (18.9%)	29 (22.3%)	60 (20.4%)	0.560^Chi^
No n (%)	133 (81.1%)	101 (77.7%)	234 (79.6%)	
*Missing values*	1 (0.6%)	1 (0.8%)	2 (0.7%)	

^a^The military personnel group includes members of the army, police, and security personnel. The independent workers group comprises drivers, miners, self-employed individuals, and construction workers. Higher education includes individuals who have completed university studies.

^b^IDR: intradermal reaction of Montenegro. The diagnosis was made based on a positive IDR, lesions suggestive of CL, and a proven epidemiological link.

^c^Species detected by PCR for Leishmania spp

^d^The following were considered risk comorbidities in patients with CL: immunosuppression and cardiovascular, respiratory, metabolic, hepatic, renal, thyroid, neoplastic diseases.

^e^The area of ulcerated lesions corresponds to the rhombus area (major diagonal x minor diagonal/2) and the area of non-ulcerated lesions corresponds to the rectangles area (base x height).

f A mixed lesion is the presence of both ulcerated and non-ulcerated lesions in the same clinical picture, in any anatomical location on the patient.

^g^The appropriate dose of MA should be based on the patient’s weight: 20 mg/kg/day, maximum 15 cc per day.

^h^The length of systemic MA for CL treatment is 20 days. Adherence to treatment means receiving complete treatment dose of 20 mg/kg/day, maximum 15 ml per day for 18 days, which means 90% of adherence. This is evaluated at the end of treatment visit when the patient brought all blister to their physician.

S1 Table shows the univariate analysis of the candidate predictors. Age (p value = 0.015) and duration of skin lesion (p value = 0.019), were significantly associated with TF. The interaction between lesion area and number of lesions was evaluated (S2 Fig). Patients presenting with more than three lesions showed an increased risk of TF as the size of the largest lesion increased, however this interaction was not significant (p-value = 0.108). Therefore, the interaction variable was not included in the multivariable model.

### Lasso model

The optimal penalty value determined by the regression method was a lambda of 0.0235 (log lambda = –3.751) with an expected shrinkage (ES) of 0.054 (log ES = –2.918) (S3 Fig). The resulting model identified the following variables as potential predictors: age, duration of skin lesion, occupation, number of lesions, and presence of local lymphadenopathy. S2 Table presents these variables along with their respective coefficients after penalization.

### Model development

**Fig 2:**
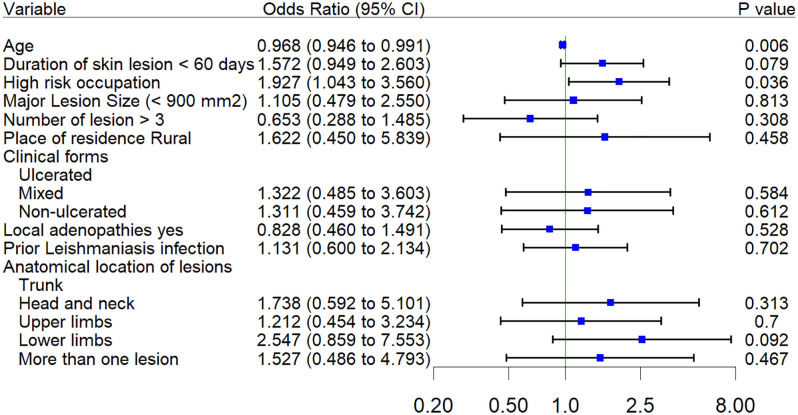
Forest plot of selected model. High-risk occupations—including military personnel and agricultural workers.

Five models were developed (S3 Table). Table 2 shows the performance of the five models. Among them, model 3 demonstrated better discrimination and calibration performance (See S4 and S5 Figs). Model 5 was chosen as the most suitable and readily applicable model. This model included ten variables: age, history of leishmaniasis, duration of skin lesion (≤ 60 days vs. > 60 days), occupation (categorized as high-risk occupations—including military personnel and agricultural workers—and low-risk occupations—including individuals with high education, independent workers such as drivers, miners, self-employed individuals, and construction workers), place of residence (urban or rural), lesion type (ulcerated, mixed, or non-ulcerated), anatomical location of lesions, number of lesions (≤ 3 lesion vs. > 3 lesions), and size of the major lesion (≤ 900 mm^2^ vs. > 900 mm^2^), in this model age (p value = 0.006), high risk occupation (p value = 0.036) were significantly related with the risk of TF. S4 Table shows the VIF of the selected model, no evidence of multicollinearity was observed among the included predictors. S4 Fig shows the Brier scores for all models, each remaining within the optimal range, indicating good overall predictive accuracy. The odds ratios, 95% CI, and p-values for the final model are presented in Fig 2 and S3 Table **(Model 5)**.

**Table 2:** Performance (calibration and discrimination) of 5 candidate models.

	HL	R^2^N	AUC/ROC (IC_95%)_	Deviance	Brier score
Model 1	0.406	0.059	0.620 (0.549 - 0.691)	334.7	23.3
Model 2	0.150	0.142	0.688 (0.620 - 0.756)	336.3	22.4
Model 3	0.767	0.171	0.705 (0.634 - 0.777)	297.3	22.6
Model 4	0.389	0.152	0.694 (0.622 - 0.765)	299.7	22.6
Model 5	0.667	0.150	0.691 (0.619 - 0.764)	297.3	22.8

HL = Hosmer and Lemeshow goodness of fit (GOF) test, R^2^N = R^2^ Nagelkerke.

**Fig 3:**
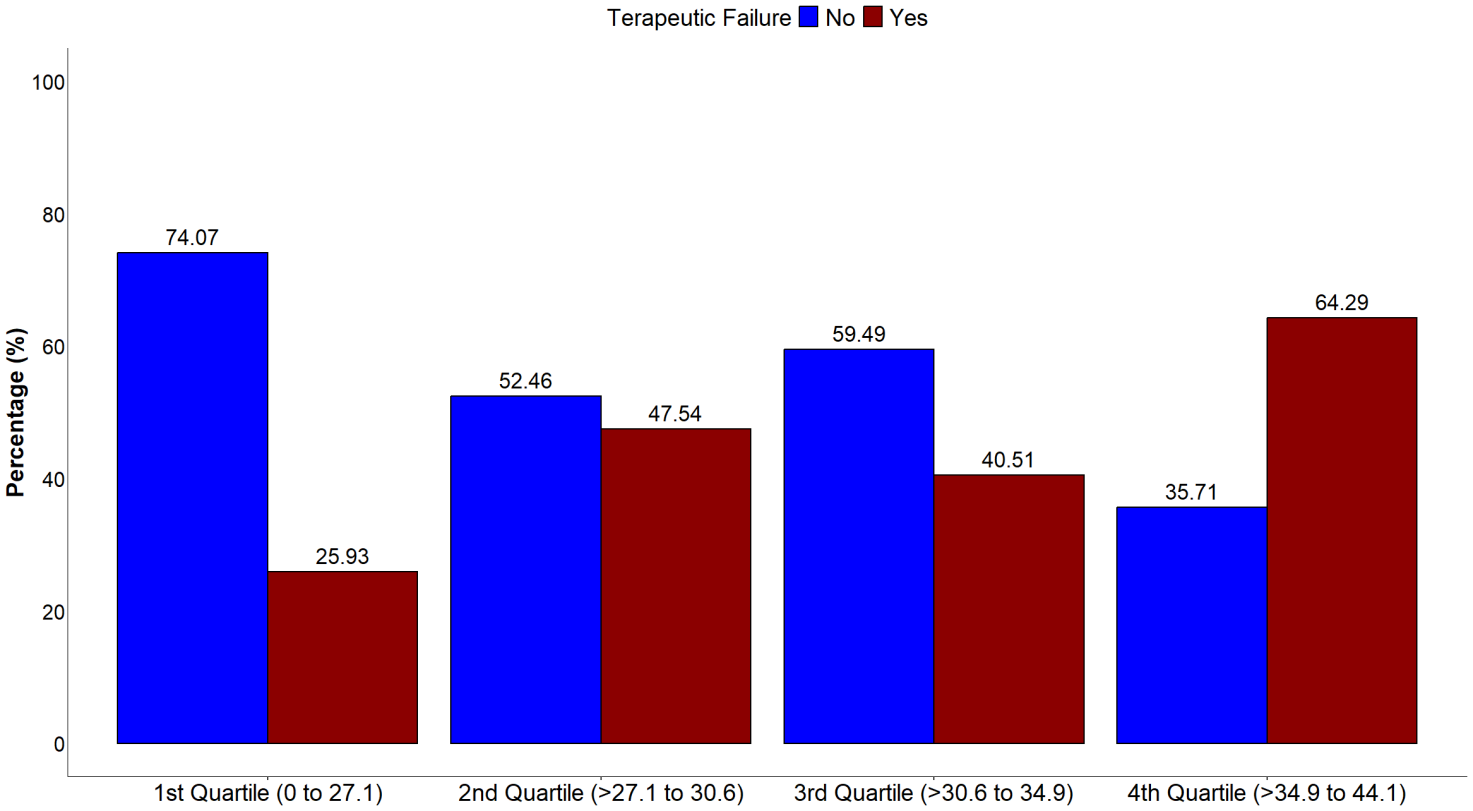
Distribution of the calculated score divided into quartiles, stratified by the presence or absence of TF.

The endpoint score was calculated using the selected multivariable model. Each variable included in the final model was assigned a weight based on its beta coefficient, which was multiplied by 10 and rounded to the nearest integer to derive the point value. The resulting scores were then categorized into quartiles to estimate the probability of FT occurrence. Within the study cohort, endpoint scores ranged from 10.6 to 44.1, with a mean of 30.4 (SD ± 6.21). As shown in Fig 3, patients with higher scores had an increased risk of TF compared to those with lower scores. Additionally, when the score was included in a logistic regression model, it significantly predicted TF risk, yielding an odds ratio (OR) of 1.105 (95% CI: 1.06–1.15; p < 0.001).

Table 3 shows sensitivity, specificity, positive predictive value (PPV), and negative predictive value (NPV) of the final model at various cutoff thresholds. At a predicted probability of 50%, the model achieved a sensitivity of 47.3% and a specificity of 72.7%, with a PPV of 57.9%. When the cutoff was lowered to below 25%, sensitivity increased at the expense of specificity, resulting in an NPV of 85.2%.

**Table 3:** Operating characteristics of the selected model.

Probability	Sens	Spec	VPP	VPN
> 25%	96.9%	13.9%	47.2%	85.2%
> 50%	47.3%	72.7%	57.9%	63.5%
> 75%	0.8%	100%	100%	55.9%

### Internal validation of the model

Internal validation yielded Harrell’s C-statistic optimism of 0.12, with an apparent C-index of 0.73 and a bias-corrected (validated) C-index of 0.61. Similarly, the optimism for the calibration slope was 0.509, with an apparent calibration slope of 1.0 and a corrected slope of 0.491.

**Fig 4:**
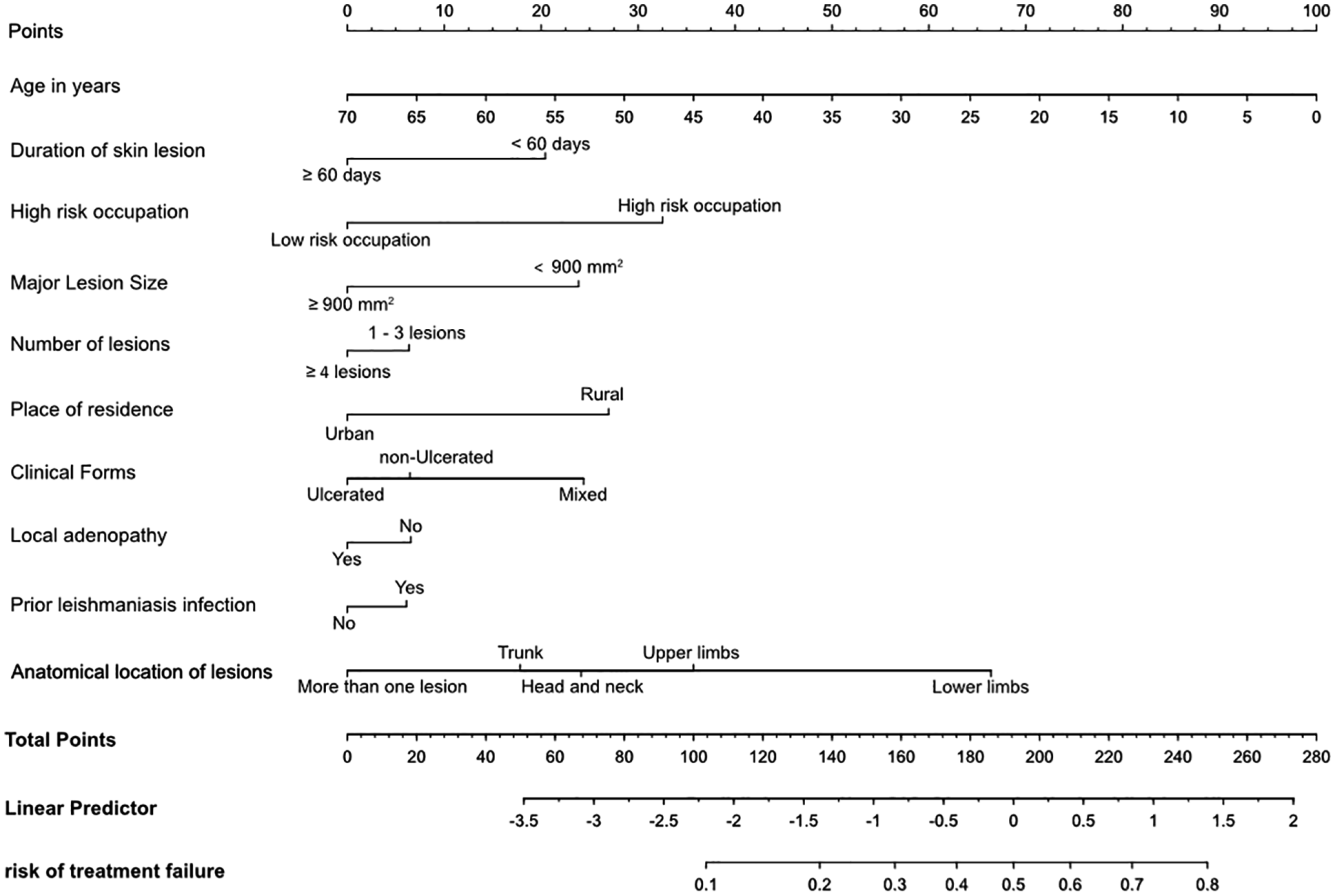
Nomogram for predicting TF. The nomogram was composed of 10 variables. The nomogram is used as follows: the first horizontal line displays the points; the reader locates how many points each predictor has according to the patient’s characteristics, and then adds up the points and locates the total number of points on the last horizontal line. Finally, the reader draws a vertical line from the total number of points and obtains the probability of TF. High-risk occupations: including military personnel and agricultural workers.

We then calculated a prediction by using a nomogram prediction. The total score of the patients was obtained by summing the scores corresponding to each subvariable to obtain the predicted probability of TF (Fig 4).

## Discussion

A clinical prediction model for TF to MA was developed using a retrospective cohort of patients with CL who received treatment at PECET, located in Medellín- Colombia. The final model includes ten easily obtainable sociodemographic and clinical variables, demonstrates moderate overall performance, acceptable discriminatory power (AUC = 0.691) and adequate calibration (Hosmer-Lemeshow test, p = 0.667). This study represents a significant advance toward risk stratification in clinical practice, particularly in resource-limited settings where molecular tests for the identification of *Leishmania* species are not routinely available.

A key finding of our study is the high incidence of TF, which reached 44.2% (n = 131) in our cohort. This number significantly exceeds the estimated prevalence of 23.4% in Latin America that was used to calculate the sample size and highlights the growing crisis in the efficacy of systemic pentavalent antimonial [2,11,13,20,35]. However, our observation is consistent with recent reports from other countries in the region that also document an increase in parasite resistance and TF rates [8,18]. This trend has been one of the main reasons for recent updates to PAHO’s CL management guidelines. Additionally, it is plausible that the high incidence of TF observed in our study is partially magnified by selection bias, given that PECET is a national referral center that tends to concentrate cases of greater clinical complexity, with longer duration and possible previous TF.

Our multivariate analysis identified predictors of TF that are consistent with both existing evidence and biological plausibility. Younger age (OR: 0.968; 95% CI: 0.946-0.991) and high-risk occupation (OR: 1.927; 95% CI: 1.043-3.560) were significantly associated with an increased risk of TF. This inverse relationship between age and TF has been previously reported [12,13,36,37]. While the precise mechanisms are not yet fully understood, evidence indicates that treatment response is multifactorial, related to differences in child immune response, drug pharmacokinetics, and antigen exposure [13,38]. Immunologically, the risk of TF in children could be due as the percentage of CD4 + T cells is lower during infancy and adolescence before rising again later in life [13,39]. CD4 + T cells are known to be important factor for Th1 proinflammatory response which drives macrophage activation, parasite clearance, and subsequent lesion healing [37,39]. Furthermore, a short exposure to parasite antigens and sand fly saliva in children may induce lower protective immunity [13,38]. Age-specific pharmacokinetic profiles in pediatric patients can explain also the risk for TF. Children exhibit a significantly higher clearance rate of AM compared to adults [40], resulting in a lower maximum plasma concentration and a reduced area under the curve [41]. These pharmacological differences highlight the critical need to improve the current clinical guidelines for pediatric CL by dose adjustment or using alternative therapeutic regimens [12].

High-risk job, which in this cohort predominantly include military personnel and agricultural workers, are associated with more intense and frequent exposure to the to the vector [18]. This occupational vulnerability is further compounded by adverse socioeconomic conditions such as poor sanitation, which led a higher incidence of FT [42,43]. Furthermore, although they did not reach statistical significance, other variables showed clinically relevant trends. Longer duration of skin lesion (OR: 1.572; p = 0.079) and location in the lower limbs (OR: 2.547; p = 0.092) emerged as important risk factors. Longer duration of skin lesion may correlate with greater parasite load, tissue fibrosis, and bacterial superinfection, factors that complicate the response to treatment [12,13,17]. Similarly, lesions in the lower extremities tend to heal more slowly due to anatomical and physiological factors, such as reduced vascularization, which has been observed in other studies as a therapeutic challenge [22].

When comparing our model with previous, the predictive model developed by Valencia et al. in Peru stands out [18]. This model achieved superior discrimination (AUC = 0.93) with an explanatory variability of 54% (R^2^ Nagelkerke = 0.54), sensitivity of 78%, and specificity of 92.5%, however, its main limitation for applicability in general clinical practice was the inclusion of *Leishmania* species identification by PCR as a key predictive variable. In many primary care and rural centers from Colombia, this technology is not available, which makes our model, based exclusively on clinical and anamnestic data, considerably more pragmatic and immediately implementable. Also, our study broadens the generalization of the findings by including patients with non-ulcerated and mixed lesions, clinical lesion type that were excluded in the Peruvian study but are associated with a higher parasite load and potentially, a higher risk of TF.

The number and size of lesions are two of the main characteristics of the infection that must be considered to make the best therapeutic decision for patients with CL [8,18,22]. Our study suggested the presence of an interaction between both variables, since in patients with more than 3 lesions, a larger lesion area increased the probability of TF. This was not observed when the number of lesions was less than 3; however, this finding did not reach statistical significance, but it is consistent with biological plausibility and supporting the recommendations to perform systemic treatment in patients with CL who have three or more lesions, at least one of which is larger than 3 cm.

The main strength of our study is its pragmatic approach: A score has been developed to be used by healthcare personnel at any level of care to estimate the risk of MA failure through a targeted medical history and physical examination. Secondly, the statistical methodology was robust, using variable selection methods such as Lasso Regression to identify the most relevant predictors and internal validation using Bootstrapping to quantify and correct model optimism, a bias inherent in the development of prognostic models. Finally, this work is aligned with PAHO guidelines [22], which advocate for a more stratified approach to CL management, considering clinical and epidemiological characteristics to guide therapeutic choices.

Our study has several limitations that must be acknowledged. First, its retrospective design makes it susceptible to information and selection biases, although strategies were implemented to minimize these, such as multiple imputations for handling missing data. Second, the model demonstrated only moderate discriminative performance, with a bias-corrected Harrell’s C-index of 0.61 after internal validation, indicating that the model has limited discriminatory power and should be considered a support tool rather than an absolute determinant of clinical decision-making. Third, the width of the 95% CI for several of the variables included in the final model suggests uncertainty in the estimates, probably due to an insufficient sample size relative to the number of predictors evaluated, which could have contributed to a certain degree of overfitting. Finally, the study cohort consists mainly of young men in the armed forces, which limits the extrapolation of the results to the female population, pediatric age groups, and other demographic contexts.

## Conclusion

This study describes the development of a predictive model for TF to systemic MA in CL, derived from routinely available clinical variables. In a scenario of TF to MA greater than 40%, the ability to identify a priori patients at higher risk of TF is crucial. Although the model demonstrated moderate performance, it remains at an early stage of development and should be interpreted with caution. External validation and evaluation in independent populations and diverse clinical settings are necessary before considering its implementation in routine practice.

Nevertheless, because the model is based on variables that are readily obtainable during a standard medical consultation and do not require specialized diagnostic tests, it may serve as a preliminary framework for future research. If validated, a risk-stratified therapeutic approach informed by such a model could potentially contribute to improving clinical decision-making, reducing unnecessary drug exposure and associated toxicity, and supporting more efficient allocation of healthcare resources in Colombia and other endemic regions

## Supporting information

S1 Fig Linear relationship to the logit of the outcome (Therapeutic failure). (DOCX)

S2 Fig Prediction of the model including interaction between number of lesions and lesion area after log transformation. (DOCX)

S3 Fig Lasso regression model (variable selection). (DOCX)

S4 Fig Calibration curves of 5 models. (DOCX)

S5 Fig ROC curve of five models. (DOCX)

S1 Table Univariate analysis (binary logistic regression). (DOCX)

S2 Table Regression coefficients after Lasso regression. (DOCX)

S3 Table Model predictions developed. (DOCX)

S4 Table Checking multicollinearity (VIF) of selected model. (DOCX)

S1 Data Description and definitions of variables in the raw dataset. (DOCX)
