# Supplementary material for: Development of a predictive model for meglumine antimoniate treatment failure in patients with cutaneous leishmaniasis: Aretrospective cohort study
Source: PLoS Negl Trop Dis. 2026 Aug 4;20(8):e0014606. doi: 10.1371/journal.pntd.0014606 (PMC13436726; doi:10.1371/journal.pntd.0014606)
Supplement: Supplementary file 3 [file pntd.0014606.s003.docx]

**S3 Fig.** Lasso Regression model (variable selection)

A

B

(A) Identification of the optimal penalization coefficient lambda (λ) in the LASSO model used 10-fold cross-validation and the minimum criterion. As a result, a λ value of 0.0235 was selected. (B) LASSO coefficient profiles of the variables evaluated
