# Supplementary material for: Development of a predictive model for meglumine antimoniate treatment failure in patients with cutaneous leishmaniasis: Aretrospective cohort study
Source: PLoS Negl Trop Dis. 2026 Aug 4;20(8):e0014606. doi: 10.1371/journal.pntd.0014606 (PMC13436726; doi:10.1371/journal.pntd.0014606)
Supplement: Supplementary file 5 [file pntd.0014606.s005.docx]

**S5 Fig:** ROC curve of five models

AUC= 0.620, IC95% (0.55-0.69)	**Model 2**	AUC= 0.688, IC_95%_(0.6200.756)

**Model 1**

**Model 3**	AUC= 0.705, IC95% (0.63-0.77)	**Model 4**	AUC= 0.694, IC_95%_ (0.62-0.76)

**Model 5**	AUC= 0.691, IC_95%_ (0.619-0.764)
