# Supplementary material for: Development of a predictive model for meglumine antimoniate treatment failure in patients with cutaneous leishmaniasis: Aretrospective cohort study
Source: PLoS Negl Trop Dis. 2026 Aug 4;20(8):e0014606. doi: 10.1371/journal.pntd.0014606 (PMC13436726; doi:10.1371/journal.pntd.0014606)
Supplement: Supplementary file 6 [file pntd.0014606.s006.docx]

**S1** **Table.** Univariate analysis (binary logistic regression)

| Variables | OR *^a^* | IC 95% *^a^* | P values |
| --- | --- | --- | --- |
| Age (years) | 0.97 | 0.95 – 0.99 | 0.015 |
| Gender Male | 1.32 | 0.67 – 2.67 | 0.40 |
| Weight | 0.99 | 0.98- 1.00 | 0.20 |
| Economic activity |  |  |  |
| Low risk |  |  |  |
| High risk^b^ | 1.48 | 0.89 – 2.51 | 0.14 |
| Comorbidities |  |  |  |
| No |  |  |  |
| Yes | 0.65 | 0.25 – 1.55 | 0.30 |
| Geographic region |  |  |  |
| Pacific | 1 |  |  |
| Amazon | 2.15 | 0.92 – 5.26 | 0.082 |
| Andean | 1.62 | 0.72 – 3.88 | 0.30 |
| Caribbean | 0.84 | 0.11 – 4.69 | 0.80 |
| Orinoquia | 1.66 | 0.66 – 4.34 | 0.30 |
| Duration of skin lesion in days *^c^*  (Log- days) | 0.66 | 0.46 – 0.93 | 0.019 |
| Clinical lesion type |  |  |  |
| Ulcerated | 1 |  |  |
| Non-ulcerated | 1.21 | 0.46 – 3.20 | 0.70 |
| Mixed | 0.74 | 0.32 – 1.61 | 0.50 |
| Number of lesions |  |  |  |
| 1 – 3 | 1 |  |  |
| 4 or more | 0.62 | 0.32 – 1.16 | 0.150 |
| Size of major lesion  (Log- mm^2^) | 0.95 | 0.82 – 1.11 | 0.60 |
| Anatomical localization of lesions |  |  |  |
| Trunk | 1 |  |  |
| Head and neck | 1.62 | 0.60 – 4.56 | 0.30 |
| Upper limbs | 1.08 | 0.43 – 2.80 | 0.90 |
| Lower limbs | 1.77 | 0.65 – 5.01 | 0.30 |
| More than one lesion | 0.99 | 0.38 – 2.72 | 0.90 |
| Presence of local adenopathy  Yes | 0.77 | 0.45 – 1.33 | 0.40 |
| Doses of AM |  |  |  |
| Insufficient | 1 |  |  |
| Sufficient | 0.30 | 0.04 – 1.41 | 0.20 |
| High doses | 0.43 | 0.05 – 2.44 | 0.40 |
| Prior Leishmaniasis infection  Yes | 1.23 | 0.69 – 2.18 | 0.50 |
| *Model including interaction between lesion area and number of lesions* | | | |
| Size of major lesion (Log- mm^2^) | 0.92 | 0.79 – 1.08 | 0.331 |
| Number of lesions (more of three) | 0.07 | 0.004 – 0.94 | 0.0621 |
| Interaction | 1.49 | 0.94 – 2.53 | 0.108 |

*^a: OR and 95% confidence intervale, b:^* ^H^*^igh-risk occupations—including military personnel and agricultural workers—and low-risk occupations—^*^including individuals with higher education,^ *^independent workers such as drivers, miners, self-employed individuals, and construction workers. Time elapsed between lesion onset and treatment initiation (in days. c:^* ^H^*^igh-risk occupations—including military personnel and agricultural workers—and low-risk occupations—^*^including individuals with higher education,^ *^independent workers such as drivers, miners, self-employed individuals, and construction workers^*
