# Supplementary material for: Development of a predictive model for meglumine antimoniate treatment failure in patients with cutaneous leishmaniasis: Aretrospective cohort study
Source: PLoS Negl Trop Dis. 2026 Aug 4;20(8):e0014606. doi: 10.1371/journal.pntd.0014606 (PMC13436726; doi:10.1371/journal.pntd.0014606)
Supplement: Supplementary file 7 [file pntd.0014606.s007.docx]

**S2 Table:** Regression Coefficients after Lasso regression

|  | **Best Lambda 0.0235** |
| --- | --- |
| **Intercept** | 0.748 |
| Age | -0.004 |
| Duration of skin lesion in days (Log) | -0.065 |
| Economic activity | 0.049 |
| Number of lesions | -0.031 |
| Local adenopathy | -0.0006 |
