# Supplementary material for: Development of a predictive model for meglumine antimoniate treatment failure in patients with cutaneous leishmaniasis: Aretrospective cohort study
Source: PLoS Negl Trop Dis. 2026 Aug 4;20(8):e0014606. doi: 10.1371/journal.pntd.0014606 (PMC13436726; doi:10.1371/journal.pntd.0014606)
Supplement: Supplementary file 8 [file pntd.0014606.s008.docx]

**S3** **Table:** Model predictions developed

|  | **Coefficient** | **SE** | **OR** | **95% CI** | **Valor p** |
| --- | --- | --- | --- | --- | --- |
| ***Model 1*** |  |  |  |  |  |
| Age | -0.026 | 0.010 | 0.974 | 0.952 - 0.994 | 0.015 |
| Duration of skin lesion in days (Log) | -0.399 | 0.178 | 0.671 | 0.468 - 0.946 | 0.026 |
| Economic activity high risk | 0.500 | 0.289 | 1.649 | 0.942 - 2.942 | 0.084 |
| Number of lesions (> 3) | -0.313 | 0.342 | 0.731 | 0.367 - 1.418 | 0.4 |
| Local adenopathy yes | -0.256 | 0.278 | 0.774 | 0.446 – 1.333 | 0.4 |
| ***Model 2*** |  |  |  |  |  |
| Age | -0.032 | 0.011 | 0.968 | 0.945 - 0.990 | 0.006 |
| Economic activity high risk | 0.482 | 0.370 | 1.619 | 0.788 - 3.386 | 0.2 |
| Duration of skin lesion in days (Log) | -0.459 | 0.187 | 0.632 | 0.433 - 0.905 | 0.014 |
| Size of major lesion (log) | 0.051 | 0.089 | 1.053 | 0.885 - 1.257 | 0.6 |
| Number of lesions (> 3) | -0.421 | 0.356 | 0.656 | 0.320 - 1.306 | 0.2 |
| Doses of AM |  |  |  |  |  |
| Subtherapeutic | -- | -- | -- | -- | -- |
| Therapeutic | -1.498 | 0.888 | 0.223 | 0.030 - 1.156 | 0.092 |
| Supratherapeutic | -0.550 | 0.987 | 0.577 | 0.067 - 3.710 | 0.6 |
| Geographic region |  |  |  |  |  |
| Pacific | -- | -- | -- | -- | -- |
| Amazon | 0.684 | 0.517 | 1.984 | 0.730 - 5.634 | 0.2 |
| Andean | 0.562 | 0.462 | 1.756 | 0.725 - 4.515 | 0.2 |
| Caribbean | 0.046 | 0.964 | 1.047 | 0.125 - 6.427 | >0.9 |
| Orinoquia | -0.573 | 0.730 | 0.563 | 0.127 - 2.319 | 0.4 |
| Prior leishmaniasis infection | 1.271 | 0.619 | 3.567 | 1.094 - 12.955 | 0.04 |
| ***Model 3*** |  |  |  |  |  |
| Age | -0.0307 | 0.011 | 0.969 | 0.946 - 0.991 | 0.008 |
| Duration of skin lesion in days (Log) | -0.372 | 0.186 | 0.678 | 0.465 - 0.971 | 0.038 |
| Economic activity high risk | 0.603 | 0.316 | 1.812 | 0.984 - 3.414 | 0.060 |
| Size of bigger lesion (log) | -0.000 | 0.096 | 1.000 | 0.827 - 1.208 | >0.9 |
| Number of lesions (> 3) | -2.098 | 1.437 | 0.123 | 0.006 - 1.730 | 0.14 |
| Place of residence Rural | 0.529 | 0.646 | 1.698 | 0.502 - 6.733 | 0.4 |
| Clinical lesion type |  |  |  |  |  |
| Ulcerated | -- | -- | -- | -- | -- |
| Non-ulcerated | 0.135 | 0.523 | 1.145 | 0.401 - 3.182 | 0.8 |
| Mixed | 0.326 | 0.527 | 1.387 | 0.489 - 3.973 | 0.5 |
| Local adenopathy yes | -0.195 | 0.294 | 0.819 | 0.457 – 1.459 | 0.5 |
| Prior leishmaniasis infection | 0.156 | 0.338 | 1.169 | 0.601 - 2.276 | 0.6 |
| Anatomical location of lesions |  |  |  |  |  |
| Trunk | -- | -- | -- | -- | -- |
| Head and neck | 0.594 | 0.549 | 1.812 | 0.624 - 5.470 | 0.3 |
| Upper limbs | 0.146 | 0.497 | 1.158 | 0.441 - 3.165 | 0.8 |
| Lower limbs | 0.922 | 0.552 | 2.515 | 0.865 - 7.647 | 0.095 |
| More than one lesion | 0.352 | 0.574 | 1.423 | 0.466 - 4.488 | 0.5 |
| Interaction: size:number of lesions | 0.328 | 0.261 | 1.388 | 0.851 -2.406 | 0.2 |
| ***Model 4*** |  |  |  |  |  |
| Age | -0.023 | 0.01 | 0.976 | 0.955 - 0.997 | 0.029 |
| Duration of skin lesion in days (Log) | -0.392 | 0.186 | 0.675 | 0.464 - 0.966 | 0.035 |
| Interaction: size:number of lesions | 0.0014 | 0.098 | 1.001 | 0.826 - 1.215 | 0.9 |
| Number of lesions (> 3) | -2.258 | 1.459 | 0.104 | 0.005 - 1.517 | 0.12 |
| Geographic region |  |  |  |  |  |
| Pacific | -- | -- | -- | -- | -- |
| Amazon | 0.766 | 0.476 | 2.153 | 0.863 - 5.662 | 0.11 |
| Andean | 0.494 | 0.452 | 1.64 | 0.689 - 4.121 | 0.3 |
| Caribbean | -0.004 | 0.973 | 0.996 | 0.117 - 6.201 | >0.9 |
| Orinoquia | -0.306 | 0.728 | 0.736 | 0.166 - 2.997 | 0.7 |
| Local adenopathy yes | -0.178 | 0.301 | 0.830 | 0.457 – 1.499 | 0.5 |
| Prior leishmaniasis infection | 1.012 | 0.613 | 2.752 | 0.854 - 9.886 | 0.1 |
| Anatomical location of lesions |  |  |  |  |  |
| Trunk | -- | -- | -- | -- | -- |
| Head and neck | 0.271 | 0.542 | 1.312 | 0.456 - 3.883 | 0.6 |
| Upper limbs | -0.043 | 0.492 | 0.957 | 0.366 - 2.579 | >0.9 |
| Lower limbs | 0.646 | 0.538 | 1.909 | 0.671 - 5.622 | 0.2 |
| More than one lesion | 0.094 | 0.554 | 1.099 | 0.373 - 3.333 | 0.9 |
| Interaction: size:number of lesions | 0.349 | 0.260 | 1.418 | 0.873 - 2.459 | 0.2 |
| ***Model 5*** |  |  |  |  |  |
| Age | -0.032 | 0.011 | 0.968 | 0.946- 0.991 | 0.006 |
| Duration of skin lesion in days < 60 days | 0.464 | 0.252 | 1.572 | 0.949- 2.603 | 0.079 |
| High risk occupation | 0.676 | 0.312 | 1.927 | 1.043- 3.560 | 0.036 |
| Major Lesion Size (< 900 mm^2^) | 0.373 | 0.421 | 1.105 | 0.479- 2.550 | 0.813 |
| Number of lesions (> 3) | -0.407 | 0.415 | 0.653 | 0.288- 1.485 | 0.308 |
| Place of residence Rural | 0.644 | 0.644 | 1.622 | 0.450- 5.839 | 0.458 |
| Clinical lesion type |  |  |  |  |  |
| Ulcerated | -- | -- | -- | -- | -- |
| Non-ulcerated | 0.441 | 0.509 | 1.322 | 0.485- 3.603 | 0.584 |
| Mixed | 0.299 | 0.533 | 1.311 | 0.459 - 3.742 | 0.612 |
| Local adenopathy yes | -0.152 | 0.293 | 0.828 | 0.460- 1.491 | 0.528 |
| Prior leishmaniasis infection | 0.102 | 0.321 | 1.131 | 0.600 - 2.134 | 0.702 |
| Anatomical location of lesions |  |  |  |  |  |
| Trunk | -- | -- | -- | -- | -- |
| Head and neck | 0.586 | 0.540 | 1.738 | 0.592- 5.101 | 0.313 |
| Upper limbs | 0.219 | 0.496 | 1.212 | 0.454- 3.234 | 0.700 |
| Lower limbs | 0.999 | 0.551 | 2.547 | 0.859- 7.553 | 0.092 |
| More than one lesion | 0.342 | 0.572 | 1.527 | 0.486- 4.793 | 0.467 |

^H^*^igh-risk occupations—including military personnel and agricultural workers—and low-risk occupations—^*^including individuals with higher education,^ *^independent workers such as drivers, miners, self-employed individuals, and construction workers^*
