# Supplementary material for: Development of a predictive model for meglumine antimoniate treatment failure in patients with cutaneous leishmaniasis: Aretrospective cohort study
Source: PLoS Negl Trop Dis. 2026 Aug 4;20(8):e0014606. doi: 10.1371/journal.pntd.0014606 (PMC13436726; doi:10.1371/journal.pntd.0014606)
Supplement: Supplementary file 9 [file pntd.0014606.s009.docx]

**S4** **Table:** Checking multicollinearity (VIF) of selected model

| **Variables** | **Inflation Variance Factor (VIF)** |
| --- | --- |
| Age | 1.107 |
| Duration of skin lesion in days < 60 days | 1.046 |
| High risk occupation | 1.295 |
| Major Lesion Size (< 900 mm^2^) | 1.070 |
| Number of lesions (> 3) | 1.037 |
| Place of residence Rural | 1.033 |
| Clinical Forms  Non-ulcerated  Mixed | 1.089  1.505 |
| Local adenopathy yes | 1.156 |
| Prior leishmaniasis infection | 1.276 |
| Anatomical location of lesions  Head and neck  Upper limbs  Lowe limbs  More than one lesion | 3.016  3.845  2.783  3.684 |

^H^*^igh-risk occupations—including military personnel and agricultural workers—and low-risk occupations—^*^including individuals with higher education,^ *^independent workers such as drivers, miners, self-employed individuals, and construction workers^*
