# Supplementary material for: Development of a predictive model for meglumine antimoniate treatment failure in patients with cutaneous leishmaniasis: Aretrospective cohort study
Source: PLoS Negl Trop Dis. 2026 Aug 4;20(8):e0014606. doi: 10.1371/journal.pntd.0014606 (PMC13436726; doi:10.1371/journal.pntd.0014606)
Supplement: Supplementary file 10 [file pntd.0014606.s010.docx]

**Explanation variables of the Dataset:**

| Variable | Explanation |
| --- | --- |
| ID | Identification number |
| Age | Age in years |
| weight | Weight at therapy initiation (kg) |
| Economic activity | Low risk: individuals with higher education, independent workers such as drivers, miners, self-employed individuals, and construction workers |
|  | High risk: military personnel and agricultural workers |
| Region | Geographical region of Colombia where the patients reside |
| Place or residence | Place of residence classified as urban or rural |
| Comorbidities | History of previous diseases: cardiovascular, respiratory, metabolic, hepatic, renal, thyroid and neoplastic diseases and use of immunosuppressive regimens |
| Leishmania PCR | Species detected by PCR for Leishmania spp |
| Diagnosis by LC | Diagnosis approaches: Direct parasitological examination or biopsy |
| Duration of skin lesion in days | Duration of skin lesion in days |
| Lesion_type | Type of cutaneous leishmaniasis lesion: ulcerated, mixed or non-ulcerated |
| Number_lesions | Number of lesions at treatment initiation |
| Area | Size of lesion, for ulcerated lesions, the area was calculated using the rhomboid formula (major diagonal × minor diagonal / 2), whereas for non-ulcerated lesions, the area was calculated using the rectangle formula (base × height). |
| Localization of the lesion | Anatomical localization of cutaneous leishmaniasis lesions |
| Head_neck | Anatomical localization of cutaneous leishmaniasis lesions |
| Trunk | Anatomical localization of cutaneous leishmaniasis lesions |
| Upper_Limbs | Anatomical localization of cutaneous leishmaniasis lesions |
| Lower_Limbs | Anatomical localization of cutaneous leishmaniasis lesions |
| Local_adenotpathy | Presence of regional lymphadenopathy |
| Adherencia_90 | Adherence to treatment was defined as receiving at least 90% of the prescribed regimen, corresponding to a complete treatment dose of 20 mg/kg/day (maximum 15 ml per day) for 18 days |
| Leishmania_previa | History of previous leishmaniasis episodes. |
| Terapeutic_Failure | Therapeutic failure systemic meglumine antimoniate, defined as the presence of at least one of the following criteria three months post treatment: 1) ≥ 50% increase in lesion size, 2) lesion progression after initial regression (including enlargement of a plaque, nodule, or ulcer), or 3) incomplete re-epithelialization and/or persistent inflammatory signs such edema or erythema |

**Ethical responsibilities**

The Ethics Committee of the Medical Research Institute of the Faculty of Medicine of the University of Antioquia of April 2024 (Acta 154-24) approved the study, and the research was performed in accordance with the Declaration of Helsinki.
