# Supplementary material for: Development of a predictive model for meglumine antimoniate treatment failure in patients with cutaneous leishmaniasis: Aretrospective cohort study
Source: PLoS Negl Trop Dis. 2026 Aug 4;20(8):e0014606. doi: 10.1371/journal.pntd.0014606 (PMC13436726; doi:10.1371/journal.pntd.0014606)
Supplement: Supplementary file 11 [file pntd.0014606.s011.docx]

Dear Dr Kazemirad, Dr Werneck, Dr Kamhawi, and Dr. Brindley

Editorial Team

PLOS Neglected Tropical diseases

We sincerely thank the reviewers for their constructive comments and valuable suggestions, which have helped us improve the quality and clarity of our manuscript. We have carefully addressed each point raised and revised the manuscript accordingly. Detailed, point-by-point responses are provided below, and all modifications have been incorporated into the revised version.

**Journal Requirements:**

1) Please provide an Author Summary. This should appear in your manuscript between the Abstract (if applicable) and the Introduction, and should be 150-200 words long. The aim should be to make your findings accessible to a wide audience that includes both scientists and non-scientists. Sample summaries can be found on our website under Submission Guidelines:

<https://journals.plos.org/plosntds/s/submission-guidelines#loc-parts-of-a-submission>

**Answer**: Thank you for this remark. An Author Summary has now been added between the Abstract and Introduction.

2) Please upload all main figures as separate Figure files tif or eps format <https://journals.plos.org/plosntds/s/figures>

**Answer**: All main figures have now been uploaded separately in TIFF format, with a maximum resolution of 2500 pixels, in accordance with journal guidelines.

3) In the online submission form, you indicated that the raw data underlying the results presented in the study are available from the PECET (Programa de Estudio y Control de Enfermedades Tropicales) for researchers who meet the criteria for access to confidential data. All PLOS journals now require all data underlying the findings described in their manuscript to be freely available to other researchers, either

- In a public repository
- Within the manuscript itself
- Uploaded as supplementary information.

This policy applies to all data except where public deposition would breach compliance with the protocol approved by your research ethics board. If your data cannot be made publicly available for ethical or legal reasons (e.g., public availability would compromise patient privacy), please explain your reasons by return email and your exemption request will be escalated to the editor for approval. Your exemption request will be handled independently and will not hold up the peer review process, but will need to be resolved should your manuscript be accepted for publication. One of the Editorial team will then be in touch if there are any issues.

**Answer:** We will accept uploaded the dataset as supplementary information

**Reviewers' Comments:**

**Methods**

**Reviewer #1:**

1) L4, aims should be stated in the past tense.

**Answer**: Corrected

2) This study considered only treatment failure cases. What happens to the relapse cases that occur after six months of follow-up assessment?

**Answer**: **Answer:** We thank the reviewer for this valuable comment. According to the *Guideline for the Treatment of Leishmaniasis in the Americas, Second Edition*, therapeutic failure is defined as the absence of clinical cure three months after completion of treatment. The appearance of a lesion at six months is not considered therapeutic failure; rather, it is classified as relapse or reinfection, which are distinct clinical outcomes. Our endpoint specifically included cases of therapeutic failure and did not include relapse or reinfection. This reference is cited in our manuscript: Pan American Health Organization (PAHO). *Guideline for the Treatment of Leishmaniasis in the Americas. Second Edition.* 2022. Available at: <https://iris.paho.org/bitstream/handle/10665.2/56120/guidelineleishmaniasisannex_eng.pdf?sequence=14>.

3) Scientific names should be written in italic font (L27, Leishmania, L28 Lutzomyia, etc.)

**Answer**: Corrected

4) Prevalently used terms should be abbreviated (L3, meglumine antimoniate; MA) and treatment failure (TF L4,5,19 and it should be defined at first appearance).

**Answer**: we thank the reviewers for this remark, we corrected the manuscript to ensure consistent first definition and appropriate abbreviation use.

5) CL was already abbreviated (L31); it should not be used in complete form /or defined (L58, 59, 276, etc.), or TF is defined in many places (L275).

**Answer**: Corrected

6) Treatment failure and case definition should be presented in M&M.

**Answer** The case definition has been clearly included in the Materials and Methods section as follows: “*The primary outcome of this study was TF to MA, defined as the presence of at least one of the following criteria three months post treatment: 1) a ≥ 50% increase in lesion size, 2) lesion progression after initial regression (including enlargement of a plaque, nodule, or ulcer), or 3) incomplete re-epithelialization and/or persistent inflammatory signs such edema or erythema (9, 25). “*

7) L83, Nagekerke is printed in small first letter?

**Answer**: Corrected

8) Since a few cases were identified to species level by PCR, how could we be sure that the remaining (n=268) are L. braziliens

**Answer:** According to our results, the species causing leishmaniasis was identified by PCR in only 28 patients. One had a confirmed infection with *L. braziliensis* and 27 with *L. panamensis*. 268 patients did not undergo PCR, so it is not possible to determine which species infected them. Additionally, given the low availability of PCR in the Latin American clinical context, it is not a feasible variable for the model, which is why this context also excluded this variable from the final model.

**Reviewer #2:**

1) Some key variables require clearer operational definitions. For example, the term “high-risk occupations” is later presented as an important finding in the results, yet the data collection section lists only “age, gender, economic activity, educational level, place of residence, and comorbidities”, without specifying how occupational risk categories were defined.

**Answer**: We agree and have now explicitly defined high-risk and low-risk occupational categories in the Methods section *“**Economic activity was categorized as high-risk occupations—including army personnel and agricultural workers—and low-risk occupations—including independent workers such as drivers, miners, self-employed individuals, and construction workers*”

2) The manuscript states that “informed consent was obtained”, but does not explain how this was feasible given the retrospective nature of the study and the extended time frame (dating back to 2007). Clarification is needed on the consent procedures, if applicable, or whether waivers were granted. Additionally, the ethics approval number is not described.

**Answer**: The study was conducted in accordance with the ethical principles established in the Declaration of Helsinki and Resolution 8430 of 1993 of the Colombian Ministry of Health. According to these guidelines, this type of research is considered risk-free. The confidentiality of participants' identifying data was guaranteed, and only the healthcare team involved in the study had access to the information. Given that the data analyzed was obtained from the medical records of patients treated at the PECET, and in accordance with the provisions of Colombian Resolution 1995 of 1999, which establishes standards for the management of medical records, the use of the data contained in them is permitted for healthcare personnel. In addition, patients or their legal representatives (in the case of minors) authorized the use of the information by completing the informed consent form (ICF) for patient care (current version for each date of care) at the research center. The different versions of the ICF had been approved by the center's safety committee. In addition to the above, the execution of the project was approved by the Medical Research Institute of the Faculty of Medicine of the University of Antioquia through Minutes 154-24 of April 2024.

3) The description of the outcome variable, particularly the timing of the three criteria used to determine treatment failure, should be improved. According to the harmonized criteria for cutaneous leishmaniasis treatment outcomes, the timing of assessments is critical (Olliaro, et al). Considering that retrospective studies often face challenges related to missing or inconsistently documented follow-up, it would be good for authors to describe how they handled variability in follow-up time points, how outcome classification was standardized, and whether any assumptions or imputation strategies were applied. A general description of imputations for missing values was presented, but it is not clear whether that also applies to the outcome measures.

**Answer:** We sincerely thank the reviewer for this careful and constructive comment. In response, we would like to clarify the approach used at PECET, which follows the *PAHO Guideline for the Treatment of Leishmaniasis in the Americas*. Accordingly, we evaluated the three criteria established in this guideline, as clearly described in the Materials and Methods section. In addition, in accordance with the PAHO recommendations and the institutional guidelines for the management of cutaneous leishmaniasis (CL) at PECET, patients are monitored using a standardized follow-up schedule. Lesions are assessed either through in-person visits or virtually (via photographs or videos). Follow-up evaluations are conducted at the end of treatment, 45 days after treatment completion, and at 3, 6, 9, and 12 months post-treatment.

Finally, we confirm that multiple imputation was performed, as detailed in the Statistical Analysis subsection of the Materials and Methods section. However the endpoint does not have missing values.

**Reviewer #3:**

1) I am not sure that this could be called a retrospective cohort study. I think it is fairer to call it a retrospective cohort analysis.

**Answer**: Corrected

**Results**

**Reviewer #1:**

1) Relapse is the follow-up assessment. A lesion following cure. Here, the authors mentioned nothing about the relapse rate post-treatment follow-up visits. The reason one visits the CL patients following treatment is to see whether the cases have relapsed or not.

**Answer:** We respectfully wish to clarify that our study focuses specifically on therapeutic failure and does not evaluate relapse. We recognize that these terms may sometimes be used interchangeably; however, they refer to distinct clinical outcomes with different definitions. As established in the *PAHO Guideline for the Treatment of Leishmaniasis in the Americas*, therapeutic failure and relapse are defined separately. Our analysis was conducted in strict accordance with these guideline definitions.

2) - It is not clear the number of variables that were significant in the result section.

**Answer**: thank you very much. We have clarified in the Results that age, lesion duration, and high-risk occupational activity were statistically significant predictors in the final model. We also clarified that model development prioritized predictive performance rather than individual variable significance alone.

**Reviewer #2:**

3) Age is presented only as mean with standard deviation. Given the well-established association between younger age and treatment outcomes in cutaneous leishmaniasis, it would be important to report the age range and explicitly indicate the proportion of pediatric patients in the cohort. This information is essential for interpreting the external validity of the findings.

**Answer**: The minimum and maximum ages, as well as the number of pediatric patients, have now been added to Table 1 and described in the Results section.

4) Table 1: Age range: Please report the minimum and maximum ages represented.

**Answer**: Corrected

5) Table 1: Economic activity: The category “professional” appears more closely related to educational attainment than to occupational classification.

**Answer:** thank you very much, we have done the correction now they are identifying as Higher education

6) Table 1: o IDR: Spell out the acronym (presumably intradermal reaction or Montenegro skin test) at first mention.

**Answer:** Yes, IDR means intradermal reaction. The definition of the acronym IDR is added to the footer of Table 1.

7) Table 1: Lesion area: The method used to calculate lesion area should be described, including the geometric formula applied.

**Answer:** thank you very much, the method to calculate the area of the lesion was included in methods follow: *The area of ulcerated lesions corresponds to the rhombus area (major diagonal x minor diagonal/2) and the area of non-ulcerated lesions corresponds to the rectangles area (base x height).*

A mixed lesion is the presence of both ulcerated and non-ulcerated lesions in the same clinical picture, in any anatomical location on the patient formation has been added to the footer of Table 1.

8) Table 1 Treatment adherence: The description requires further detail. For example, reporting “18/20 doses” is insufficient without specifying the timeframe (e.g., over how many days) and how adherence was assessed relative to prescribed dosing. Although retrospective designs impose limitations on quality of adherence data, clarity regarding dose administration patterns is important given the short elimination half-life of antimonials and their potential sensitivity to gaps between doses.

**Answer:** information has been updated and clarified at the footer of Table 1 and methods section.

9) Table 1 Lesion duration: Kindly check whether the time unit is months or days. Although chronic lesions can persist for extended periods, a median duration of five years (60 months) would contradict existing literature.

**Answer:** you are right, thank you for the remark, there was a mistake. Those are days not months.

10) Line 174: The “endpoint score” is introduced without any explanation in the Methods. The authors should clarify what this score represents and whether it was pre-specified or created post hoc. 3. Line 174:

**Answer**: We thank the reviewers for this remark. The endpoint score was calculated using the selected multivariable model. Each variable included in the final model was assigned a weight based on its beta coefficient, which was multiplied by 10 and rounded to the nearest integer to derive the point value. The resulting scores were then categorized into quartiles to estimate the probability of FT occurrence. We have added this information to the manuscript in results and methods sections.

11) Although the authors describe the modeling steps thoroughly, the clinical meaning of the results is insufficiently addressed. Several included predictors are not defined, making interpretation difficult. Examples include High-risk occupation: The term is not defined.

**Answer**: High risk occupation now is defined.

12) Non-local adenopathy: The rationale for using this category instead of standard regional adenopathy is unclear.

**Answer**: this was an error in the forest plot labeling. The correct term is **local adenopathy yes**. We have corrected this in the figure and throughout the manuscript.

13) Of the ten predictors in the final model, only age and high-risk occupation were significant, but their clinical implications are not discussed in depth. The supplementary figures help clarify some associations, but this is not reflected in the main text.

**Answer:** Thank you for your comment, We have already discussed the implications of age and high-risk professions in the discussion section.

**Minor corrections:**

1) 1. Kindly check the most appropriate term in the first paragraph (patients or records), since it is a study with retrospective evaluation of electronic health data. Are all the records from one PECET or multiple facilities? If multiple facilities, kindly indicate whether clinical management practices were comparable across sites.

**Answer:** Thank you for this important comment. All clinical records were obtained from a single center, PECET (Programa de Estudio y Control de Enfermedades Tropicales), Universidad de Antioquia, Medellín, Colombia. This is the only PECET institution in the country. We have clarified this in the Methods section of the manuscript.

2) The model development details may be more appropriate in the Methods. As currently written, they break the flow of the Results.

**Answer**: Thank you very much for this helpful suggestion. We have carefully reviewed the manuscript and moved all methodological information that was previously presented in the Results section to the Methods section to improve clarity and structure

**Reviewer #3**

3) The internal validation results sections are very short and simple. Since this is announced in the title as one of the strengths of this study, this section should be larger and more detailed. Another option is to remove this information from the title.

**Answer**: Thank you for this important comment. We have removed the word “internal validation” from the title, as the model is still in an exploratory phase and requires validation in independent cohorts to confirm the stability and relevance of the identified predictors.

**Conclusions**

**Reviewer #1 Suitable**

**Reviewer #2 Suitable**

1) The discussion summarizes the main findings, and the text is well written. My main concern is, however, that the clinical use and interpretation of the finding is limited. It is important that authors explicitly address how these findings could be used in clinical settings and provide stronger justification for the selected variables. Some relevant points are elaborated below:

**Answer:** thank you very much, the information was updated in the discussion section.

2) The authors attribute the association between younger age and treatment failure primarily to host immunity. However, other well-known explanations such as age-related differences in antimonial pharmacokinetics, should be acknowledged. In general, providing a more balanced interpretation would strengthen this section.

**Answer:** information updated at discussion

3) 2. In addition, greater clarity on the age distribution would help readers understand the observed patterns. The supplementary figure suggests a U-shaped risk curve, with wider confidence intervals at age extremes, likely due to small sample sizes. The discussion should state how many very young and very old participants were included, as this can affect the interpretation of the finding.

**Answer**: Thank you for this valuable comment. The minimum and maximum ages, along with the number of pediatric participants in the cohort, have now been added to Table 1 and described in the Results section.

4) The lesion duration is inconsistent across tables. It is unclear whether lesion duration was recorded in days or months.

**Answer:** it has been updated, in days not months.

5) Some tables and figures appear inconsistent. This needs verification, since this affects interpretation and the plausibility of the results.

**Answer**: Thank you for this important comment. We have thoroughly reviewed all tables and figures to ensure consistency and accuracy and have corrected the identified errors and typographical issues.

6). The final statement claims that the model provides a “valuable tool” to optimize management, yet the manuscript does not explain how it could be used in practice. Beyond a broad statement, the discussion does not elaborate on how clinicians could apply the model. Only two of the ten predictors were statistically significant, and the clinical utility of the remaining variables is not addressed. Given the model’s moderate performance and lack of external validation, the conclusions should be more cautious and specify the steps needed before clinical implementation.

**Answer**: Thank you for this thoughtful and important comment. We agree that the model remains exploratory and should not be presented as ready for clinical implementation. In response, we have revised the Discussion and Conclusion sections to moderate our statements and clearly emphasize that the model requires external validation in independent cohorts and evaluation in diverse clinical settings before being considered for routine clinical use.

**Reviewer #3:**

1) The authors should moderate their optimism about the potential use of the predictive model in clinical practice. As they recognize, the model has limitations because of its moderate overall performance and moderate sensitivity and specificity. On the other hand, 10 variables could be considered a very large number of variables to be assessed by a clinician with each patient. Also, before considering the potential use of any model, it needs to be operationalized and adapted to be the clinical practice settings. The authors should expand a little bit more this aspect in the discussion and conclusion.

**Answer**: Thank you for this thoughtful and important comment. We agree that the model remains exploratory and should not be presented as ready for clinical implementation. In response, we have revised the Discussion and Conclusion sections to moderate our statements and clearly emphasize that the model requires external validation in independent cohorts and evaluation in diverse clinical settings before being considered for routine clinical use.

2) On the other hand, 10 variables could be considered a very large number of variables to be assessed by a clinician with each patient. Also, before considering the potential use of any model, it needs to be operationalized and adapted to be the clinical practice settings. The authors should expand a little bit more this aspect in the discussion and conclusion.

**Answer:** it has been updated,

**Editorial and Data Presentation Modifications?**

**Reviewer #1:**

1) The manuscript is well prepared, clinically relevant, fairly reliable methods and appropriate modeling approaches (multivariate logistic regression). Some concerns about the referral center cases that are mixed (it is a solitary center retrospective cohort study). I believe the manuscript can be suitable for publication if the authors clearly complete and respond to the above questions. There are two types of unresponsiveness to treatment: 1- treatment failure, which is the cases that do not respond to the assigned drug basically; 2- Resistant cases are those that initially respond to the action of the drug and get cured but later on recur, and that is why, 6 months following treatment, they should be re-examined and managed with other drugs. The authors ignored such relapsed cases.

**Answer:** Yes, we only work with therapeutic failures. Our approach did not cover the period after the third month of follow-up treatment. Then we talk about a relapse, but we did not evaluate that; we evaluated therapeutic failure.

**Reviewer #2:**

Comments are described across the review text for each section.

**Reviewer #3:**

1)  There are editorial aspects that need to be reviewed. There are some missing words and inconsistency in the use of some abbreviations

**Answer**: Thank you for this important comment. We have thoroughly revised the manuscript to correct typographical errors, address missing words, and ensure consistent use of abbreviations and terminology throughout.

**Summary and General Comments**

**Reviewer #2:**

1) The manuscript addresses a relevant topic and presents a dataset with potential to inform clinical practice. However, the interpretation of the results appear insufficiently developed in their current form. The Results and Discussion sections rely heavily on statistical descriptions, with limited attention to the clinical meaning of the findings, variable definitions, and practical implications of the model. These gaps make it difficult to fully assess the model’s potential and the contribution it brings to the existing literature. With additional clarification and more emphasis on interpretation, the work could become a more solid contribution, but substantial revision is needed before its strengths can be fully appreciated

**Answer:** thank you, information has been updated at discussion and conclusion according to this recommendation so clinical potential has been added.

**Reviewer #3:**

This is a very interesting article, based on a study conducted by a research group with extensive experience in cutaneous leishmaniasis research, resulting in one of the best clinical datasets on this disease in the region. The development and validation of models that can predict glucantime treatment failure, and that can be used by primary care personnel in endemic areas, is crucial, given that this is a neglected tropical disease and that laboratory resources are very limited for evaluating patients and predicting treatment failure. This study contributes to that goal.

Answer: thank you very much for your evaluation.
