# Supplementary material for: Development of a predictive model for meglumine antimoniate treatment failure in patients with cutaneous leishmaniasis: Aretrospective cohort study
Source: PLoS Negl Trop Dis. 2026 Aug 4;20(8):e0014606. doi: 10.1371/journal.pntd.0014606 (PMC13436726; doi:10.1371/journal.pntd.0014606)
Supplement: Supplementary file 12 [file pntd.0014606.s012.docx]

Dear Dr Kazemirad, Dr Werneck, Dr Kamhawi, and Dr. Brindley

Editorial Team

PLOS Neglected Tropical diseases

We sincerely thank the reviewers for their constructive comments and valuable suggestions. We have carefully addressed each point raised and revised the manuscript accordingly. Detailed, point-by-point responses are provided below, and all modifications have been incorporated into the revised version.

**Reviewers' Comments:**

**Methods**

**Reviewer #2:**

The methods are overall clear and aligned with the study aims. Some recommendations to improve this section include**:**

1) Lines 107-108: please consider adding a citation to support the use of Rubin’s Rules for pooling estimates after multiple imputation. There also appears to be a minor typo in this sentence: “the estimated were pooled”, which should read “the estimates were pooled.”

**Answer**: we thank the reviewers for this remark, we added two citations to support the use of Rubin´s Rules. We have also corrected the typo. (Line 150)

2) The statement “All participants provided written informed consent” may be misleading in the context of a retrospective study, as it could be interpreted as consent specifically for participation in this study. Based on the authors’ response and the manuscript context, it appears that patients provided consent for medical care and possibly for secondary use of their clinical data. Please revise the wording to clarify the nature and scope of the consent obtained, and distinguish this from study-specific informed consent, if applicable.

**Answer**: thank you very much, indeed, participants provide consent for medical care and for secondary use of the clinical data the informed consent form for leishmaniasis medical consultation, which is signed at the PECET as a standardized process, so they are free to choose whether their clinical data can be used in clinical research. I have improved this paragraph. (Lines 100 – 109).

**Reviewer #3:**

3) Yes. The study objective is clearly articulated with the study results. Authors improved this section after the first revision and took into account the reviewer comments.

**Answer**: thank you very much for you comment.

**Results**

**Reviewer #2:**

4) The Results section is clear, and the presentation has improved. In particular, removing some of the model development text from this section has improved readability. However, some additional comments remain, mostly related to proofreading and clarity of writing rather than to the quality of the study. More broadly, the text may benefit from proofreading by a native English speaker or a professional language editing service.

**Answer**: Thank you for the note. We have carefully revised the manuscript and corrected all English language errors.

5) Lines 118 – 120: “When comparing patients with and without TF, differences were found in terms of occupation, with professionals having higher cure rates (p=0.06) and lower adherence to therapy (less than 90%).” This sentence is difficult to interpret and would benefit from revision. In its current form, it is unclear whether the higher cure rates and lower adherence to therapy refer to professionals or to patients with TF. Please clarify the comparison being made and the direction of the reported differences.

**Answer:** We sincerely thank the reviewer for this comment. We have improved the redaction of this sentence to make interpretable. (Lines 163 – 165)

6) 204: Leishmania should be italicized.

**Answer**: Thank you for the comment. We have revised the manuscript and italicized *Leishmania* throughout the text.

7) Figure 2: Please note the term “evolution time” is not commonly used in English in this context and may reflect a direct translation from Spanish. Consider revising it to a more standard term, such as “disease duration,” “duration of symptoms,” or “time since symptom onset,” depending on the intended meaning.

A similar observation applies to the phrase “localization of single lesion”. Authors may consider revising it to “location of single lesion,” “site of single lesion,” or “anatomical location of single lesion.”

**Answer:** Thank you for this comment. We have corrected the manuscript for consistency. "Evolution time" has been updated to "duration of skin lesion," and "localization of lesions" has been changed to "anatomical location of lesions." (Line 224)

**Reviewer #3:**

8) Yes. The results are clearly and completely presented. Tables and figures are adequate.

**Answer**: thank you very much

**Conclusion**

**Reviewer #2:**

The Discussion and Conclusion sections are generally clear and aligned with the data presented in the results; however, some statements would benefit from stronger grounding in the existing literature and further clarification. Comments and suggestions to improve these sections are listed below.

9) Authors state that “PECET is a national referral center that tends to concentrate cases of greater clinical complexity, with longer duration and possible previous TF.” Please clarify whether this was observed in the study population. Specifically, how many participants had documented previous therapeutic failure, and how might this have influenced the results and the interpretation of the model?

**Answer:** Thank you for the comment. In the discussion section, we address the high incidence of therapeutic failure (TF) in our study (44.2%), which was higher than the historical regional estimate of 23.4%. This discrepancy is primarily due to our institution's status as a national referral center, which usually includes more complex cases. Specifically, a documented history of previous infection was present in 60 participants (20.4%), of whom 29 (22.3% of the subgroup) experienced therapeutic failure.

Regarding the multivariable model, several factors must be considered during interpretation. First, due the high incidence of therapeutic failure rate to 44.2%, the model's predicted might be overestimated. Second, the variable *prior Leishmania infection* did not reach statistical significance Odds Ratio: 1.131; p = 0.702), suggesting that the biological impact of drug resistance might be diluted within this specific model. Consequently, we have framed this model as a preliminary, supportive tool rather than an absolute determinant of clinical decision-making.

10) Lines 217–219: “The association with age has been previously reported, although the underlying mechanisms, possibly related to the host immune response, are not yet fully understood.”

Please note that this issue, raised in the previous review, does not appear to have been adequately addressed. The association between age and therapeutic response to antileishmanial treatment has been reported in multiple studies, and several hypotheses have been proposed to explain this relationship. However, the revised text still provides a broad statement about host immune response without citing the relevant literature or engaging with other proposed explanations. Please revise this section to include appropriate citations and a more complete discussion of the possible mechanisms underlying the association with age

**Answer**: we thank the reviewers for this remark, we added more information related age and the risk of FT. (Lines 291 - 308)

11) In the conclusion, the claim that this is “the first model on the continent” appears overstated and potentially inaccurate. A prior model from Peru is already mentioned in the Discussion, which would seem to preclude describing the present study in this way. Please revise the statement to avoid overclaiming novelty and to clearly distinguish the contribution of this study from previously published work.

**Answer**: corrected. (Line 364)

12) The clinical applicability of the proposed score remains unclear. The model includes many variables, 10 in total, yet the manuscript does not explain how clinicians are expected to calculate, interpret, or act on the score. In addition, given that the therapeutic failure rate in this cohort is higher than that reported in other settings in the region, please discuss how this may affect the generalizability of the score, particularly in populations with different baseline risks.

**Answer**: corrected. We have added a nomogram, which shows the probability of treatment failure based the variables included in the multivariable model. (Lines 253 - 268)

13) Minor comment: Line 220: The term “militaries,” when referring to individuals in high-risk occupations, should be revised. Please consider using alternatives such as “military personnel,” “service members,” or “members of the armed forces,” as appropriate.

**Answer**: corrected.

**Summary and General Comments**

Reviewer #2: Overall, the manuscript has improved, particularly in the presentation of the Results section and the readability of the model development description. However, I recommend further revision. The Discussion and Conclusion sections still require additional work, particularly to better ground key statements in the existing literature, clarify the interpretation and applicability of the proposed model, and address concerns regarding generalizability and clinical use of the score. I would also recommend proofreading by a native English speaker or copyediting services.

Reviewer #3: Although not many people care about treatment failure in cutaneous leishmaniasis, this is a big problem for patients. Being a neglected disease, it is important for them and the doctors and nurses who are responsible of treating these patients and be aware of clinical and epidemiological patient characteristics that could predict treatment failure. Therefore, this paper is valuable. Further work is needed to validate and improve this model, but this is a good start.
