# Supplementary material for: Development of a predictive model for meglumine antimoniate treatment failure in patients with cutaneous leishmaniasis: Aretrospective cohort study
Source: PLoS Negl Trop Dis. 2026 Aug 4;20(8):e0014606. doi: 10.1371/journal.pntd.0014606 (PMC13436726; doi:10.1371/journal.pntd.0014606)
Supplement: Peer review history

# Peer review: Decision Letter 0

**DOI:** 10.1371/journal.pntd.0014606.r001

**Authors:** Guilherme L Werneck, Elham Kazemirad

1 Feb 2026

PNTD-D-25-02094

Development and Internal Validation of a Predictive Model for Meglumine Antimoniate Treatment Failure in Patients with Cutaneous Leishmaniasis: A Retrospective Cohort Study

PLOS Neglected Tropical Diseases

Dear Dr. Serna-Higuita,

Thank you for submitting your manuscript to PLOS Neglected Tropical Diseases. After careful consideration, we feel that it has merit but does not fully meet PLOS Neglected Tropical Diseases's publication criteria as it currently stands. Therefore, we invite you to submit a revised version of the manuscript that addresses the points raised during the review process.

Please submit your revised manuscript within by Apr 02 2026 11:59PM. If you will need more time than this to complete your revisions, please reply to this message or contact the journal office at plosntds@plos.org. When you're ready to submit your revision, log on to https://www.editorialmanager.com/pntd/ and select the 'Submissions Needing Revision' folder to locate your manuscript file.

Please include the following items when submitting your revised manuscript:

* A letter that responds to each point raised by the editor and reviewer(s). You should upload this letter as a separate file labeled 'Response to Reviewers'. This file does not need to include responses to any formatting updates and technical items listed in the 'Journal Requirements' section below.

* A marked-up copy of your manuscript that highlights changes made to the original version. You should upload this as a separate file labeled 'Revised Manuscript with Track Changes'.

* An unmarked version of your revised paper without tracked changes. You should upload this as a separate file labeled 'Manuscript'.

If you would like to make changes to your financial disclosure, competing interests statement, or data availability statement, please make these updates within the submission form at the time of resubmission. Guidelines for resubmitting your figure files are available below the reviewer comments at the end of this letter.

We look forward to receiving your revised manuscript.

Kind regards,

Elham Kazemirad, Ph.D

Academic Editor

PLOS Neglected Tropical Diseases

Guilherme Werneck

Section Editor

PLOS Neglected Tropical Diseases

Shaden Kamhawi

co-Editor-in-Chief

PLOS Neglected Tropical Diseases

orcid.org/0000-0003-4304-636XX

Paul Brindley

co-Editor-in-Chief

PLOS Neglected Tropical Diseases

orcid.org/0000-0003-1765-0002

**Journal Requirements:**

If the reviewer comments include a recommendation to cite specific previously published works, please review and evaluate these publications to determine whether they are relevant and should be cited. There is no requirement to cite these works unless the editor has indicated otherwise.

1) Please provide an Author Summary. This should appear in your manuscript between the Abstract (if applicable) and the Introduction, and should be 150-200 words long. The aim should be to make your findings accessible to a wide audience that includes both scientists and non-scientists. Sample summaries can be found on our website under Submission Guidelines:

https://journals.plos.org/plosntds/s/submission-guidelines#loc-parts-of-a-submission

2) Please upload all main figures as separate Figure files in .tif or .eps format. For more information about how to convert and format your figure files please see our guidelines:

https://journals.plos.org/plosntds/s/figures

3) In the online submission form, you indicated that The raw data underlying the results presented in the study are available from the PECET (Programa de Estudio y Control de Enfermedades Tropicales) for researchers who meet the criteria for access to confidential data.. All PLOS journals now require all data underlying the findings described in their manuscript to be freely available to other researchers, either

1. In a public repository

2. Within the manuscript itself

3. Uploaded as supplementary information.

This policy applies to all data except where public deposition would breach compliance with the protocol approved by your research ethics board. If your data cannot be made publicly available for ethical or legal reasons (e.g., public availability would compromise patient privacy), please explain your reasons by return email and your exemption request will be escalated to the editor for approval. Your exemption request will be handled independently and will not hold up the peer review process, but will need to be resolved should your manuscript be accepted for publication. One of the Editorial team will then be in touch if there are any issues.

**Reviewers' Comments:**

Reviewer's Responses to Questions

**Key Review Criteria Required for Acceptance?**

As you describe the new analyses required for acceptance, please consider the following:

**Methods**

-Are the objectives of the study clearly articulated with a clear testable hypothesis stated?

-Is the study design appropriate to address the stated objectives?

-Is the population clearly described and appropriate for the hypothesis being tested?

-Is the sample size sufficient to ensure adequate power to address the hypothesis being tested?

-Were correct statistical analysis used to support conclusions?

-Are there concerns about ethical or regulatory requirements being met?

Reviewer #1: - L4, aims should be stated in the past tense.

- This study considered only treatment failure cases. What happens to the relapse cases that occur after six months of follow-up assessment?

- Scientific names should be written in italic font (L27, Leishmania, L28 Lutzomyia, etc.)

- Prevalently used terms should be abbreviated (L3, meglumine antimoniate; MA) and treatment failure (TF L4,5,19 and it should be defined at first appearance).

- CL was already abbreviated (L31); it should not be used in complete form /or defined (L58, 59, 276, etc.), or TF is defined in many places (L275).

- Treatment failure and case definition should be presented in M&M.

- L83, Nagekerke is printed in small first letter?

- Since a few cases were identified to species level by PCR, how could we be sure that the remaining (n=268) are L. braziliens?

Reviewer #2: Overall, the methodological section is clearly presented. The study objectives are explicitly stated, and both the design and the analytic strategy appear appropriate for addressing the research aims. The population is generally well described, and the study setting seems adequate for testing the hypotheses posed. Nevertheless, some methodological aspects would benefit from clarification or further detail:

1. Some key variables require clearer operational definitions. For example, the term “high-risk occupations” is later presented as an important finding in the results, yet the data collection section lists only “age, gender, economic activity, educational level, place of residence, and comorbidities”, without specifying how occupational risk categories were defined.

2. The manuscript states that “informed consent was obtained”, but does not explain how this was feasible given the retrospective nature of the study and the extended time frame (dating back to 2007). Clarification is needed on the consent procedures, if applicable, or whether waivers were granted. Additionally, the ethics approval number is not described.

3. The description of the outcome variable, particularly the timing of the three criteria used to determine treatment failure, should be improved. According to the harmonized criteria for cutaneous leishmaniasis treatment outcomes, the timing of assessments is critical (Olliaro, et al). Considering that retrospective studies often face challenges related to missing or inconsistently documented follow-up, it would be good for authors to describe how they handled variability in follow-up time points, how outcome classification was standardized, and whether any assumptions or imputation strategies were applied. A general description of imputations for missing values was presented, but it is not clear whether that also applies to the outcome measures.

Reviewer #3: I am not sure that this could be called a retrospective cohort study. I think it is more fair to call it a retrospective cohort analysis.

**********

**Results**

-Does the analysis presented match the analysis plan?

-Are the results clearly and completely presented?

-Are the figures (Tables, Images) of sufficient quality for clarity?

Reviewer #1: Results are alright. - Treatment failure cases were patients who demonstrated active lesion(s) or relapse at a defined period (6 months) of follow-up examination.

- Relapse is the follow-up assessment. A lesion following cure. Here, the authors mentioned nothing about the relapse rate post-treatment follow-up visits. The reason one visits the CL patients following treatment is to see whether the cases have relapsed or not.

- It is not clear the number of variables that were significant in the result section.

Reviewer #2: The results are generally aligned with the analytical approach described in the methods, and the figures and tables are of good quality. Below I list some aspects that require clarification to improve clarity and interpretation of the findings.

1. Age is presented only as a mean with standard deviation. Given the well-established association between younger age and treatment outcomes in cutaneous leishmaniasis, it would be important to report the age range and explicitly indicate the proportion of pediatric patients in the cohort. This information is essential for interpreting the external validity of the findings.

2. Table 1:

o Age range: Please report the minimum and maximum ages represented.

o Economic activity: The category “professional” appears more closely related to educational attainment than to occupational classification.

o IDR: Spell out the acronym (presumably intradermal reaction or Montenegro skin test) at first mention.

o Lesion area: The method used to calculate lesion area should be described, including the geometric formula applied.

o Treatment adherence: The description requires further detail. For example, reporting “18/20 doses” is insufficient without specifying the timeframe (e.g., over how many days) and how adherence was assessed relative to prescribed dosing. Although retrospective designs impose limitations on quality f adherence data, clarity regarding dose administration patterns is important given the short elimination half-life of antimonials and their potential sensitivity to gaps between doses.

o Lesion duration: Kindly check whether the time unit is months or days. Although chronic lesions can persist for extended periods, a median duration of five years (60 months) would contradict existing literature.

3. Line 174: The “endpoint score” is introduced without any explanation in the Methods. The authors should clarify what this score represents and whether it was pre-specified or created post hoc.

4. Although the authors describe the modeling steps thoroughly, the clinical meaning of the results is insufficiently addressed. Several included predictors are not defined, making interpretation difficult. Examples include:

o High-risk occupation: The term is not defined.

o Non-local adenopathy: The rationale for using this category instead of standard regional adenopathy is unclear.

Of the ten predictors in the final model, only age and high-risk occupation were significant, but their clinical implications are not discussed in depth. The supplementary figures help clarify some associations, but this is not reflected in the main text.

Minor comments:

1. Kindly check the most appropriate term in the first paragraph (patients or records), since it is a study with retrospective evaluation of electronic health data. Are all the records from one PECET or multiple facilities? If multiple facilities, kindly indicate whether clinical management practices were comparable across sites.

2. The model development details may be more appropriate in the Methods. As currently written, they break the flow of the Results.

Reviewer #3: The internal validation results sections is very short and simple. Since this is announced in the title as one of the strengths of this study, this section should be larger and more detailed. Another option is to remove this information from the title.

**********

**Conclusions**

-Are the conclusions supported by the data presented?

-Are the limitations of analysis clearly described?

-Do the authors discuss how these data can be helpful to advance our understanding of the topic under study?

-Is public health relevance addressed?

Reviewer #1: Suitable.

Reviewer #2: The discussion summarizes the main findings, and the text is overall well written. My main concern is, however, that the clinical use and interpretation of the finding is limited. It is important that authors explicitly address how these findings could be used in clinical settings and provide stronger justification for the selected variables. Some relevant points are elaborated below:

1. The authors attribute the association between younger age and treatment failure primarily to host immunity. However, other well-known explanations such as age-related differences in antimonial pharmacokinetics, should be acknowledged. In general, providing a more balanced interpretation would strengthen this section.

2. In addition, greater clarity on the age distribution would help readers understand the observed patterns. The supplementary figure suggests a U-shaped risk curve, with wider confidence intervals at age extremes, likely due to small sample sizes. The discussion should state how many very young and very old participants were included, as this can affect the interpretation of the finding.

3. The lesion duration is inconsistent across tables. It is unclear whether lesion duration was recorded in days or months. Some tables and figures appear inconsistent. This needs verification, since this affects interpretation and the plausibility of the results.

4. The final statement claims that the model provides a “valuable tool” to optimize management, yet the manuscript does not explain how it could be used in practice. Beyond a broad statement, the discussion does not elaborate on how clinicians could apply the model. Only two of the ten predictors were statistically significant, and the clinical utility of the remaining variables is not addressed.

Given the model’s moderate performance and lack of external validation, the conclusions should be more cautious and specify the steps needed before clinical implementation.

Reviewer #3: The authors should moderate their optimism about the potential use of the predictive model in clinical practice. As they recognize, the model has limitations because of its moderate overall performance and moderate sensitivity and specificity. On the other hand, 10 variables could be considered a very large number of variables to be assessed by a clinician with each patient. Also, before considering the potential use of any model, it needs to be operationalized and adapted to be the clinical practice settings. The authors should expand a little bit more this aspect in the discussion and conclusion.

**********

**Editorial and Data Presentation Modifications?**

Use this section for editorial suggestions as well as relatively minor modifications of existing data that would enhance clarity. If the only modifications needed are minor and/or editorial, you may wish to recommend “Minor Revision” or “Accept”.

Reviewer #1: The manuscript is well prepared, clinically relevant, fairly reliable methods and appropriate modeling approaches (multivariate logistic regression). Some concerns about the referral center cases that are mixed (it is a solitary center retrospective cohort study). I believe the manuscript can be suitable for publication if the authors clearly complete and respond to the above questions. There are two types of unresponsiveness to treatment: 1- treatment failure, which is the cases that do not respond to the assigned drug basically; 2- Resistant cases are those that initially respond to the action of the drug and get cured but later on recur, and that is why, 6 months following treatment, they should be re-examined and managed with other drugs. The authors ignored such relapsed cases.

Reviewer #2: Comments are described across the review text for each section.

Reviewer #3: There are editorial aspects that need to be reviewed. There are some missing words and inconsistency in the use of some abbreviations.

**********

**Summary and General Comments**

Use this section to provide overall comments, discuss strengths/weaknesses of the study, novelty, significance, general execution and scholarship. You may also include additional comments for the author, including concerns about dual publication, research ethics, or publication ethics. If requesting major revision, please articulate the new experiments that are needed.

Reviewer #1: (No Response)

Reviewer #2: The manuscript addresses a relevant topic and presents a dataset with potential to inform clinical practice. However, the interpretation of the results appear insufficiently developed in their current form. The Results and Discussion sections rely heavily on statistical descriptions, with limited attention to the clinical meaning of the findings, variable definitions, and practical implications of the model. These gaps make it difficult to fully assess the model’s potential and the contribution it brings to the existing literature. With additional clarification and more emphasis on interpretation, the work could become a more solid contribution, but substantial revision is needed before its strengths can be fully appreciated

Reviewer #3: This is a very interesting article, based on a study conducted by a research group with extensive experience in cutaneous leishmaniasis research, resulting in one of the best clinical datasets on this disease in the region. The development and validation of models that can predict glucantime treatment failure, and that can be used by primary care personnel in endemic areas, is crucial, given that this is a neglected tropical disease and that laboratory resources are very limited for evaluating patients and predicting treatment failure. This study contributes to that goal.

**********

PLOS authors have the option to publish the peer review history of their article (what does this mean?). If published, this will include your full peer review and any attached files.

If you choose “no”, your identity will remain anonymous but your review may still be made public.

**Do you want your identity to be public for this peer review?**  For information about this choice, including consent withdrawal, please see our Privacy Policy.

Reviewer #1: No

Reviewer #2: No

Reviewer #3: No

**Figure resubmission:**

While revising your submission, we strongly recommend that you use PLOS’s NAAS tool (https://ngplosjournals.pagemajik.ai/artanalysis) to test your figure files. NAAS can convert your figure files to the TIFF file type and meet basic requirements (such as print size, resolution), or provide you with a report on issues that do not meet our requirements and that NAAS cannot fix.

After uploading your figures to PLOS’s NAAS tool - https://ngplosjournals.pagemajik.ai/artanalysis, NAAS will process the files provided and display the results in the "Uploaded Files" section of the page as the processing is complete. If the uploaded figures meet our requirements (or NAAS is able to fix the files to meet our requirements), the figure will be marked as "fixed" above. If NAAS is unable to fix the files, a red "failed" label will appear above. When NAAS has confirmed that the figure files meet our requirements, please download the file via the download option, and include these NAAS processed figure files when submitting your revised manuscript.

**Reproducibility:**

To enhance the reproducibility of your results, we recommend that authors of applicable studies deposit laboratory protocols in protocols.io, where a protocol can be assigned its own identifier (DOI) such that it can be cited independently in the future. Additionally, PLOS ONE offers an option to publish peer-reviewed clinical study protocols. Read more information on sharing protocols at https://plos.org/protocols?utm_medium=editorial-email&utm_source=authorletters&utm_campaign=protocols

# Author comment: Author response to Decision Letter 1

**DOI:** 10.1371/journal.pntd.0014606.r002

16 Mar 2026

Attachment Submitted filename: Answer Letter S-R_LMSH 24-FEB-26.docx

# Peer review: Decision Letter 1

**DOI:** 10.1371/journal.pntd.0014606.r003

**Authors:** Guilherme L Werneck, Elham Kazemirad

8 Jun 2026

PNTD-D-25-02094R1

Development of a Predictive Model for Meglumine Antimoniate Treatment Failure in Patients with Cutaneous Leishmaniasis: A Retrospective Cohort Study

PLOS Neglected Tropical Diseases

Dear Dr. Serna-Higuita,

Thank you for submitting your manuscript to PLOS Neglected Tropical Diseases. After careful consideration, we feel that it has merit but does not fully meet PLOS Neglected Tropical Diseases's publication criteria as it currently stands. Therefore, we invite you to submit a revised version of the manuscript that addresses the points raised during the review process.

Please submit your revised manuscript by Jul 08 2026 11:59PM. If you will need more time than this to complete your revisions, please reply to this message or contact the journal office at plosntds@plos.org. When you're ready to submit your revision, log on to https://www.editorialmanager.com/pntd/ and select the 'Submissions Needing Revision' folder to locate your manuscript file.

Please include the following items when submitting your revised manuscript:

* A letter that responds to each point raised by the editor and reviewer(s). You should upload this letter as a separate file labeled 'Response to Reviewers'. This file does not need to include responses to any formatting updates and technical items listed in the 'Journal Requirements' section below.

* A marked-up copy of your manuscript that highlights changes made to the original version. You should upload this as a separate file labeled 'Revised Manuscript with Track Changes'.

* An unmarked version of your revised paper without tracked changes. You should upload this as a separate file labeled 'Manuscript'.

If you would like to make changes to your financial disclosure, competing interests statement, or data availability statement, please make these updates within the submission form at the time of resubmission. Guidelines for resubmitting your figure files are available below the reviewer comments at the end of this letter.

As the corresponding author, your ORCID iD is verified in the submission system and will appear in the published article. PLOS supports the use of ORCID, and we encourage all coauthors to register for an ORCID iD and use it as well. Please encourage your coauthors to verify their ORCID iD within the submission system before final acceptance, as unverified ORCID iDs will not appear in the published article. Only the individual author can complete the verification step; PLOS staff cannot verify ORCID iDs on behalf of authors.

We look forward to receiving your revised manuscript.

Kind regards,

Elham Kazemirad, Ph.D

Academic Editor

PLOS Neglected Tropical Diseases

Guilherme Werneck

Section Editor

PLOS Neglected Tropical Diseases

Shaden Kamhawi

co-Editor-in-Chief

PLOS Neglected Tropical Diseases

orcid.org/0000-0003-4304-636XX

Paul Brindley

co-Editor-in-Chief

PLOS Neglected Tropical Diseases

orcid.org/0000-0003-1765-0002

**Additional Editor Comments (if provided):**

**Journal Requirements:**

If the reviewer comments include a recommendation to cite specific previously published works, please review and evaluate these publications to determine whether they are relevant and should be cited. There is no requirement to cite these works unless the editor has indicated otherwise.

1) Please upload all main figures as separate Figure files in .tif or .eps format. For more information about how to convert and format your figure files please see our guidelines:

https://journals.plos.org/plosntds/s/figures.

**Reviewers' comments:**

Reviewer's Responses to Questions

**Key Review Criteria Required for Acceptance?**

As you describe the new analyses required for acceptance, please consider the following:

**Methods**

-Are the objectives of the study clearly articulated with a clear testable hypothesis stated?

-Is the study design appropriate to address the stated objectives?

-Is the population clearly described and appropriate for the hypothesis being tested?

-Is the sample size sufficient to ensure adequate power to address the hypothesis being tested?

-Were correct statistical analysis used to support conclusions?

-Are there concerns about ethical or regulatory requirements being met?

Reviewer #2: The methods are overall clear and aligned with the study aims. Some recommendations to improve this section include:

Lines 107-108: please consider adding a citation to support the use of Rubin’s Rules for pooling estimates after multiple imputation. There also appears to be a minor typo in this sentence: “the estimated were pooled”, which should read “the estimates were pooled.”

The statement “All participants provided written informed consent” may be misleading in the context of a retrospective study, as it could be interpreted as consent specifically for participation in this study. Based on the authors’ response and the manuscript context, it appears that patients provided consent for medical care and possibly for secondary use of their clinical data. Please revise the wording to clarify the nature and scope of the consent obtained, and distinguish this from study-specific informed consent, if applicable.

Reviewer #3: Yes. The study objetive is clearly articulated with the study results. Authors improved this section after the first revision and took into account the reviewer comments.

**********

**Results**

-Does the analysis presented match the analysis plan?

-Are the results clearly and completely presented?

-Are the figures (Tables, Images) of sufficient quality for clarity?

Reviewer #2: The Results section is clear, and the presentation has improved. In particular, removing some of the model development text from this section has improved readability. However, some additional comments remain, mostly related to proofreading and clarity of writing rather than to the quality of the study. More broadly, the text may benefit from proofreading by a native English speaker or a professional language editing service.

Some of the comments are listed below:

Lines 118 – 120: “When comparing patients with and without TF, differences were found in terms of occupation, with professionals having higher cure rates (p=0.06) and lower adherence to therapy (less than 90%).”

This sentence is difficult to interpret and would benefit from revision. In its current form, it is unclear whether the higher cure rates and lower adherence to therapy refer to professionals or to patients with TF. Please clarify the comparison being made and the direction of the reported differences.

204: Leishmania should be italicized.

Figure 2: Please note the term “evolution time” is not commonly used in English in this context and may reflect a direct translation from Spanish. Consider revising it to a more standard term, such as “disease duration,” “duration of symptoms,” or “time since symptom onset,” depending on the intended meaning.

A similar observation applies to the phrase “localization of single lesion”. Authors may consider revising it to “location of single lesion,” “site of single lesion,” or “anatomical location of single lesion.”

Reviewer #3: Yes. The results are clearly and completely presented. Tables and figures are adequate.

**********

**Conclusions**

-Are the conclusions supported by the data presented?

-Are the limitations of analysis clearly described?

-Do the authors discuss how these data can be helpful to advance our understanding of the topic under study?

-Is public health relevance addressed?

Reviewer #2: The Discussion and Conclusion sections are generally clear and aligned with the data presented in the results; however, some statements would benefit from stronger grounding in the existing literature and further clarification. Comments and suggestions to improve these sections are listed below.

Authors state that “PECET is a national referral center that tends to concentrate cases of greater clinical complexity, with longer duration and possible previous TF.” Please clarify whether this was observed in the study population. Specifically, how many participants had documented previous therapeutic failure, and how might this have influenced the results and the interpretation of the model?

Lines 217–219: “The association with age has been previously reported, although the underlying mechanisms, possibly related to the host immune response, are not yet fully understood.”

Please note that this issue, raised in the previous review, does not appear to have been adequately addressed. The association between age and therapeutic response to antileishmanial treatment has been reported in multiple studies, and several hypotheses have been proposed to explain this relationship. However, the revised text still provides a broad statement about host immune response without citing the relevant literature or engaging with other proposed explanations. Please revise this section to include appropriate citations and a more complete discussion of the possible mechanisms underlying the association with age.

In the conclusion, the claim that this is “the first model on the continent” appears overstated and potentially inaccurate. A prior model from Peru is already mentioned in the Discussion, which would seem to preclude describing the present study in this way. Please revise the statement to avoid overclaiming novelty and to clearly distinguish the contribution of this study from previously published work.

The clinical applicability of the proposed score remains unclear. The model includes many variables, 10 in total, yet the manuscript does not explain how clinicians are expected to calculate, interpret, or act on the score. In addition, given that the therapeutic failure rate in this cohort is higher than that reported in other settings in the region, please discuss how this may affect the generalizability of the score, particularly in populations with different baseline risks.

Minor comment: Line 220: The term “militaries,” when referring to individuals in high-risk occupations, should be revised. Please consider using alternatives such as “military personnel,” “service members,” or “members of the armed forces,” as appropriate.

Reviewer #3: Yes.

**********

**Editorial and Data Presentation Modifications?**

Use this section for editorial suggestions as well as relatively minor modifications of existing data that would enhance clarity. If the only modifications needed are minor and/or editorial, you may wish to recommend “Minor Revision” or “Accept”.

Reviewer #2: (No Response)

Reviewer #3: No further comments.

**********

**Summary and General Comments**

Use this section to provide overall comments, discuss strengths/weaknesses of the study, novelty, significance, general execution and scholarship. You may also include additional comments for the author, including concerns about dual publication, research ethics, or publication ethics. If requesting major revision, please articulate the new experiments that are needed.

Reviewer #2: Overall, the manuscript has improved, particularly in the presentation of the Results section and the readability of the model development description. However, I recommend further revision. The Discussion and Conclusion sections still require additional work, particularly to better ground key statements in the existing literature, clarify the interpretation and applicability of the proposed model, and address concerns regarding generalizability and clinical use of the score. I would also recommend proofreading by a native English speaker or copyediting services.

Reviewer #3: Although not many people care about treatment failure in cutaneous leishmaniasis, this is a big problem for patients. Being a neglected disease, it is important for them and the doctors and nurses who are responsible of treating this patients and be aware of clinical and epidemiological patient characteristics that could predict treatment failure. Therefore, this paper is valuable. Further work is needed to validate and improve this model, but this is a good start.

**********

PLOS authors have the option to publish the peer review history of their article (what does this mean?). If published, this will include your full peer review and any attached files.

If you choose “no”, your identity will remain anonymous but your review may still be made public.

**Do you want your identity to be public for this peer review?**  For information about this choice, including consent withdrawal, please see our Privacy Policy.

Reviewer #2: No

Reviewer #3: No

[NOTE: If reviewer comments were submitted as an attachment file, they will be attached to this email and accessible via the submission site. Please log into your account, locate the manuscript record, and check for the action link "View Attachments". If this link does not appear, there are no attachment files.]

**Figure resubmission:**

While revising your submission, we strongly recommend that you use PLOS’s NAAS tool (https://ngplosjournals.pagemajik.ai/artanalysis) to test your figure files. NAAS can convert your figure files to the TIFF file type and meet basic requirements (such as print size, resolution), or provide you with a report on issues that do not meet our requirements and that NAAS cannot fix.

After uploading your figures to PLOS’s NAAS tool - https://ngplosjournals.pagemajik.ai/artanalysis, NAAS will process the files provided and display the results in the "Uploaded Files" section of the page as the processing is complete. If the uploaded figures meet our requirements (or NAAS is able to fix the files to meet our requirements), the figure will be marked as "fixed" above. If NAAS is unable to fix the files, a red "failed" label will appear above. When NAAS has confirmed that the figure files meet our requirements, please download the file via the download option, and include these NAAS processed figure files when submitting your revised manuscript.

**Reproducibility:**

To enhance the reproducibility of your results, we recommend that authors of applicable studies deposit laboratory protocols in protocols.io, where a protocol can be assigned its own identifier (DOI) such that it can be cited independently in the future. Additionally, PLOS ONE offers an option to publish peer-reviewed clinical study protocols. Read more information on sharing protocols at https://plos.org/protocols?utm_medium=editorial-email&utm_source=authorletters&utm_campaign=protocols

# Author comment: Author response to Decision Letter 2

**DOI:** 10.1371/journal.pntd.0014606.r004

17 Jun 2026

Attachment Submitted filename: Answer Letter 16-Jun-26.docx

# Peer review: Decision Letter 2

**DOI:** 10.1371/journal.pntd.0014606.r005

**Authors:** Guilherme L Werneck

22 Jul 2026

Dear Dr Serna-Higuita,

We are pleased to inform you that your manuscript 'Development of a Predictive Model for Meglumine Antimoniate Treatment Failure in Patients with Cutaneous Leishmaniasis: A Retrospective Cohort Study' has been provisionally accepted for publication in PLOS Neglected Tropical Diseases.

Before your manuscript can be formally accepted you will need to complete some formatting changes, which you will receive in a follow up email. A member of our team will be in touch with a set of requests.

Please note that your manuscript will not be scheduled for publication until you have made the required changes, so a swift response is appreciated.

IMPORTANT: The editorial review process is now complete. PLOS will only permit corrections to spelling, formatting or significant scientific errors from this point onwards. Requests for major changes, or any which affect the scientific understanding of your work, will cause delays to the publication date of your manuscript.

Should you, your institution's press office or the journal office choose to press release your paper, you will automatically be opted out of early publication. We ask that you notify us now if you or your institution is planning to press release the article. All press must be co-ordinated with PLOS.

Thank you again for supporting Open Access publishing; we are looking forward to publishing your work in PLOS Neglected Tropical Diseases.

Best regards,

Guilherme Werneck

Section Editor

PLOS Neglected Tropical Diseases

Shaden Kamhawi

co-Editor-in-Chief

PLOS Neglected Tropical Diseases

orcid.org/0000-0003-4304-636XX

Paul Brindley

co-Editor-in-Chief

PLOS Neglected Tropical Diseases

orcid.org/0000-0003-1765-0002

***********************************************************

p.p1 {margin: 0.0px 0.0px 0.0px 0.0px; line-height: 16.0px; font: 14.0px Arial; color: #323333; -webkit-text-stroke: #323333}span.s1 {font-kerning: none

Reviewer's Responses to Questions

**Key Review Criteria Required for Acceptance?**

As you describe the new analyses required for acceptance, please consider the following:

**Methods**

-Are the objectives of the study clearly articulated with a clear testable hypothesis stated?

-Is the study design appropriate to address the stated objectives?

-Is the population clearly described and appropriate for the hypothesis being tested?

-Is the sample size sufficient to ensure adequate power to address the hypothesis being tested?

-Were correct statistical analysis used to support conclusions?

-Are there concerns about ethical or regulatory requirements being met?

Reviewer #2: (No Response)

**********

**Results**

-Does the analysis presented match the analysis plan?

-Are the results clearly and completely presented?

-Are the figures (Tables, Images) of sufficient quality for clarity?

Reviewer #2: (No Response)

**********

**Conclusions**

-Are the conclusions supported by the data presented?

-Are the limitations of analysis clearly described?

-Do the authors discuss how these data can be helpful to advance our understanding of the topic under study?

-Is public health relevance addressed?

Reviewer #2: (No Response)

**********

**Editorial and Data Presentation Modifications?**

Use this section for editorial suggestions as well as relatively minor modifications of existing data that would enhance clarity. If the only modifications needed are minor and/or editorial, you may wish to recommend “Minor Revision” or “Accept”.

Reviewer #2: (No Response)

**********

**Summary and General Comments**

Use this section to provide overall comments, discuss strengths/weaknesses of the study, novelty, significance, general execution and scholarship. You may also include additional comments for the author, including concerns about dual publication, research ethics, or publication ethics. If requesting major revision, please articulate the new experiments that are needed.

Reviewer #2: The manuscript is clear, and its overall quality has improved. The authors have addressed the reviewers’ comments, particularly through revisions to the Discussion and Conclusions sections. I recommend a final proofread to correct the remaining minor typographical errors throughout the text.

**********

PLOS authors have the option to publish the peer review history of their article (what does this mean?). If published, this will include your full peer review and any attached files.

If you choose “no”, your identity will remain anonymous but your review may still be made public.

**Do you want your identity to be public for this peer review?**  For information about this choice, including consent withdrawal, please see our Privacy Policy.

Reviewer #2: No

# Editor report: Acceptance letter

**DOI:** 10.1371/journal.pntd.0014606.r006

**Authors:** Guilherme L Werneck

Dear Dr Serna-Higuita,

We are delighted to inform you that your manuscript, "Development of a Predictive Model for Meglumine Antimoniate Treatment Failure in Patients with Cutaneous Leishmaniasis: A Retrospective Cohort Study," has been formally accepted for publication in PLOS Neglected Tropical Diseases.

We have now passed your article onto the PLOS Production Department who will complete the rest of the publication process. All authors will receive a confirmation email upon publication.

The corresponding author will soon be receiving a typeset proof for review, to ensure errors have not been introduced during production. Please review the PDF proof of your manuscript carefully, as this is the last chance to correct any scientific or type-setting errors. Please note that major changes, or those which affect the scientific understanding of the work, will likely cause delays to the publication date of your manuscript. Note: Proofs for Front Matter articles (Editorial, Viewpoint, Symposium, Review, etc...) are generated on a different schedule and may not be made available as quickly.

Soon after your final files are uploaded, the early version of your manuscript will be published online unless you opted out of this process. The date of the early version will be your article's publication date. The final article will be published to the same URL, and all versions of the paper will be accessible to readers.

For Research Articles, you will receive an invoice from PLOS for your publication fee after your manuscript has reached the completed accept phase. If you receive an email requesting payment before acceptance or for any other service, this may be a phishing scheme. Learn how to identify phishing emails and protect your accounts at https://explore.plos.org/phishing.

Thank you again for supporting open-access publishing; we are looking forward to publishing your work in PLOS Neglected Tropical Diseases.

Best regards,

Shaden Kamhawi

co-Editor-in-Chief

PLOS Neglected Tropical Diseases

Paul Brindley

co-Editor-in-Chief

PLOS Neglected Tropical Diseases
